# Therapeutic Potential of Tea and Its Compounds in the Central Nervous System Disorders

**DOI:** 10.3390/nu18213512

**Published:** 2026-10-25

**Authors:** Joanna Dragan, Ewa Koziorowska-Gawron, Marianna Pietkiewicz, Anna Pokryszko-Dragan

**Affiliations:** 1 Division of Clinical Chemistry and Laboratory Haematology, Department of Medical Laboratory Diagnostics, Faculty of Pharmacy, Wroclaw Medical University, Borowska 211a, 50-556 Wrocław, Poland; 2 Clinical Department of Neurology, University Centre of Neurology and Neurosurgery, Faculty of Medicine, Wroclaw Medical University, Borowska 213, 50-556 Wrocław, Poland; ewa.koziorowska-gawron@umw.edu.pl (E.K.-G.); anna.pokryszko-dragan@umw.edu.pl (A.P.-D.); 3 Faculty of Pharmacy, Wroclaw Medical University, Borowska 211, 50-556 Wrocław, Poland; marianna.pietkiewicz@student.umw.edu.pl

**Keywords:** tea, polyphenols, central nervous system disorders, prevention, treatment

## Abstract

Due to increasing prevalence and burden of the central nervous system (CNS) disorders and challenges associated with their treatment, there is ongoing search for new therapeutic targets, frequently conducted in the area of natural products. Tea (derived from *Camellia sinensis*) contains a wide range of bioactive compounds (polyphenols, alkaloids and amino acids) which positively affect general health and function of many systems, including CNS. The aim of this narrative review was to present up-to-date evidence of beneficial effects of tea and its compounds on CNS functions and to outline the most promising perspectives for their utility in management of neurological conditions. Based on extensive literature search, the authors demonstrate biochemical properties of tea-derived substances, molecular mechanisms of their antioxidative, anti-inflammatory and cytoprotective activity, as well as pathways of their specific impact on CNS. The evidence is presented from experimental and clinical studies investigating effects of tea and its compounds on the prevention or amelioration of neurodegenerative disorders (Alzheimer’s disease, Parkinson’s disease, Huntington’s disease), multiple sclerosis, cerebrovascular disease and brain tumors. Finally, limitations and barriers to these investigations are discussed and their emerging future directions are suggested.

## 1. Introduction

Since its legendary origins in China over 4000 years ago, throughout the centuries, tea has become one of the most popular beverages in the world consumed every day. It is derived from the leaves, stems and buds of the plant *Camellia sinensis* (*Theaceae*) grown in China and India. Depending on the type of fermentation and further processing of the plant parts, which affect oxidation degree of their compounds, a few types of tea are distinguished (yellow, white, green, black, oolong and Pu-erh), which differ in chemical characteristics and biological properties. Regular use of tea, cultivated in many countries and traditions, is not only associated with its taste quality and positive impact on general well-being, but also with its potential health benefits [1,2].

Tea contains a wide range of bioactive compounds, including a major proportion of polyphenols (e.g., catechins, flavones, phenolic acids), as well as amino acids (L-theanine) and alkaloids (caffeine). Due to their chemical structure and metabolic profile, polyphenols (particularly abundant in green tea) exhibit strong antioxidant and anti-inflammatory properties, which have been linked to their beneficial effect on the cardiovascular system function and metabolic balance (control of weight, regulation of glucose metabolism, hepatoprotection). Modulation of immune response (e.g., anti-microbial activity) and anti-cancer effects have also been postulated as health benefits of tea compounds. Moreover, polyphenols combined with L-theanine and caffeine were suggested to have a positive impact upon nervous system function, including enhancement of cognitive performance, emotional stability and reduction in stress [3,4,5,6,7].

The incidence and burden of central nervous system (CNS) diseases in general population are constantly increasing due to aging of contemporary societies and adverse effects of environmental factors. Neurological conditions have become the leading cause of mortality and disability worldwide. Their profound impact upon patients’ functioning and quality of life, as well as burden on the healthcare system, contribute to significant clinical and social problems [8]. In spite of recent progress in understanding the background of many CNS disorders, followed by development of diagnostic and therapeutic opportunities, some of their aspects still pose a challenge to researchers and clinicians. This refers in particular to neurodegenerative diseases, with treatment options often limited to symptomatic relief, without effective control of their initiation and progression [9,10,11]. Thus, there is an ongoing extensive search for new biomarkers and therapeutic targets, frequently conducted in the area of natural compounds. Furthermore, in line with complex approach to management of CNS disorders, modification of lifestyle factors (including dietary habits) is promoted for their prevention and as a complement to pharmacological treatment. There is an increasing amount of evidence from relevant investigations which indeed demonstrates beneficial properties of tea and its compounds with regard to CNS disorders [6,7].

The aim of this study is to review up-to-date evidence of beneficial effects of tea and its compounds on CNS functions and to outline the most promising perspectives for their utility in management of neurological conditions.

## 2. Method—Literature Search

A literature search was conducted using PubMed, Scopus, Google Scholar and Cochrane Central Register of Controlled Trials databases, within the period from the beginning of 2010 until May 31, 2026. A combination of the following key terms (adjusted according to the database specifications) was used to search the relevant records: „tea intake”, „tea compounds”, “central nervous system” and “diseases/disorders”. Following the initial selection, the papers written in languages other than English and conference abstracts were excluded, and duplicates were removed. The retrieved papers were analyzed for their relevance to the topic, and further potentially relevant papers were identified in the reference lists of these texts. The literature search was primarily conducted by the first author, with the results reviewed, discussed and verified by the other authors. Finally, 224 published studies were considered the most relevant to the topic of review and included in the reference list.

## 3. Tea Compounds and Their Properties

A few types of tea are distinguished, which are obtained through different manufacturing regimens, including particular stages of plant parts (leaves, stems or buds) processing (Table 1). The final product in the form of powder, teabags or loose leaves, is available for brewing to prepare a beverage with desired strength and taste qualities [12].

Tea contains many bioactive compounds, with a major contribution of polyphenols. This group comprises: catechins (epicatechin—EC, epigallocatechin—EGC, epicatechin gallate—ECG, epigallocatechin gallate—EGCG), theaflavins (the products of the enzymatic oxidation of catechins), flavonols and their glycosides (kaempferol, quercetin), flavones, and phenolic acids (gallic and ellagic acid). Other substances contained in tea include alkaloids (caffeine) and amino acids (L-theanine) [13,14,15].

Particular stages of tea manufacturing contribute to varying oxidation degrees of its compounds and result in different contents of particular compounds in different tea types. E.g. the amount of catechins is the highest in green tea and the amounts of caffeine and theaflavins are the highest in black tea, while other types do not contain theaflavins at all (Table 1) [4,16].

The bioavailability of tea compounds is affected by the temperature and brewing time. For catechins, optimal brewing conditions are achieved at 85 °C for 3 min, when their concentration reaches its maximum level. Prolonged heating diminishes the subsequent absorption of catechins and also negatively affects tea taste and aroma [17,18]. Measurements performed for EGCG demonstrated that its peak plasma concentration is reached within 1–2 h after oral administration and ultimately decreases within 24 h. However, only up to 1.15% of the compounds contained in each ingested tea portion ends up in systemic circulation [19]. Such low bioavailability and membrane permeability result, i.a., from reduced stability in alkaline media during digestion and high susceptibility to metabolic transformations, including oxidative degradation [20].

### 3.1. Molecular Mechanisms of Tea Compounds Bioactivities

**Figure 1:**
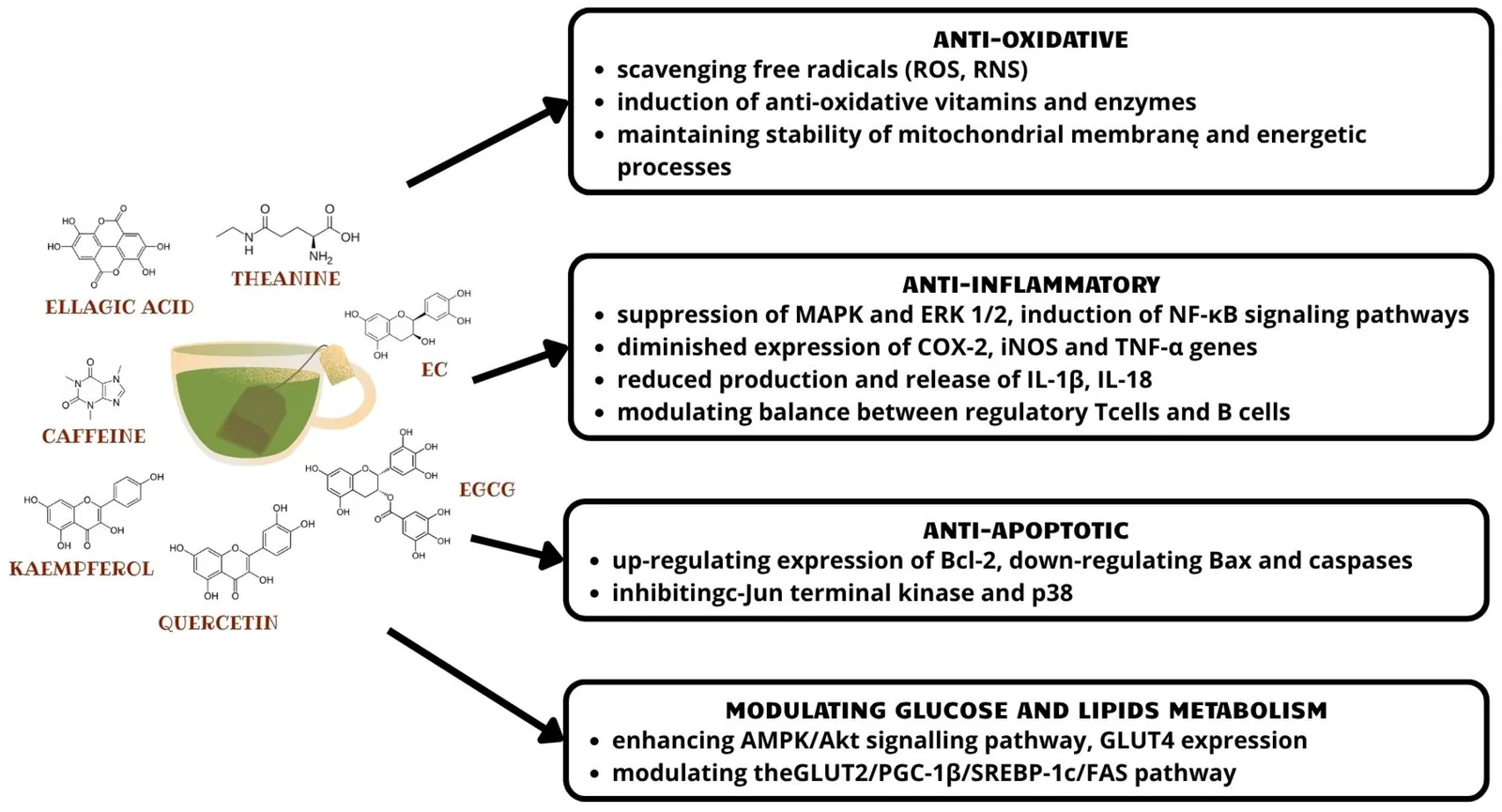
Molecular mechanisms of tea compounds bioactivity. Abbreviations: EC—epicatechin; EGCG—epigallocatechin gallate.

Tea compounds are engaged in a range of cellular defensive mechanisms, aimed at prevention or neutralization of intrinsic and extrinsic detrimental factors. These mechanisms are associated with direct chemical interactions, modulating expression of particular genes, as well as affecting multiple signaling pathways (Figure 1).

Polyphenols, especially catechins, display relevant antioxidant activity due to their chemical structure. Aromatic rings and hydroxyl groups facilitate electron delocalization, which allows them to neutralize reactive oxygen species (ROS), reactive nitrogen species (RNS) and other free radicals, as well as enables their scavenging [6,21]. Effects of polyphenols also include induction of antioxidant vitamins and enzymes (e.g., superoxide dismutase, catalase and glutathione peroxidase) and suppression of pro-oxidative ones. Apart from polyphenols, caffeine may also contribute to the antioxidant activity of tea [12]. Due to these properties, tea compounds can prevent the initiation of lipid and protein peroxidation and emerging oxidative stress-mediated cellular injury. Furthermore, polyphenols contribute to maintaining mitochondrial membrane integrity, stabilizing cellular energetic processes [22].

Tea compounds have also demonstrated considerable anti-inflammatory potential involving multiple mechanisms. Catechins, theaflavins and (to a lesser extent) caffeine suppress activation of the mitogen-activated protein kinase (MAPK) and extracellular signal-regulated kinases (ERK 1/2) cascade, while inducing nuclear factor-κB (NF-κB) signaling pathway. Consequently, transcription and expression of pro-inflammatory genes (cyclooxygenase 2—COX-2, inducible nitric oxide synthase -iNOS and tumor necrosis factor α—TNF-α) are diminished. EGCG was also shown to attenuate the activation of the NOD-like receptor family pyrin domain-containing 3 (NLRP3) inflammasome, leading to reduced production of the pro-inflammatory interleukins 1 β and 18 (IL-1β, IL-18). Furthermore, catechins can modulate balance between regulatory T cells and B cells, restoring immune homeostasis and (together with L-theanine) preventing the release of pro-inflammatory cytokines from immunocompetent cells [7,22].

By exerting antioxidant and anti-inflammatory effects, tea polyphenols can promote cell survival and prevent apoptosis in healthy tissues. Furthermore, they modulate expression of proteins, shifting the balance towards anti-apoptotic (Bcl-2) proteins and downregulating pro-apoptotic ones (Bax and caspase-3). They may also inhibit enzymes (c-Jun terminal kinase and p38) which play an important role in intracellular apoptotic signaling. On the contrary, catechins were demonstrated to induce apoptosis selectively on various cancer cell lines, playing a gene regulatory role analogous to that of p53 protein (e.g., enhancing expression of caspases and downregulating expression of sirtuin 1). These properties of catechins, parallel to their effects on cell proliferation (altering the expression of cell cycle regulatory proteins, i.a. Bax, C-Myc, Bcl-2) and modulation of signal transduction pathway (activity of p27 and mammalian target of rapamycin mTOR)—contribute to anti-cancer potential of these tea compounds [4,7,23].

Another aspect of tea compound activity is associated with regulation of cellular metabolic processes, mainly by modulating glucose and lipid metabolism and improving insulin sensitivity. Catechins, as well as L-theanine, have been shown to promote anabolic processes through activating the AMP-activated protein kinase/Protein Kinase B (AMPK/Akt) signaling pathway and enhancing glucose transporter protein (GLUT4) expression. As a result, there is an increase in glucose cellular uptake, triggered by exercise or insulin, and an increase in lipolytic enzymes activity. In addition, catechins may suppress gluconeogenesis and lipogenesis by modulating the GLUT2/PGC-1β/SREBP-1c/FAS pathway, preventing excessive lipid accumulation and insulin resistance. Influence of caffeine upon glucose metabolism is controversial (depending on dose and short or long-term effects) but it was consistently demonstrated to stimulate fatty acids turnover and lipid oxidation [4,24,25].

Thanks to these properties of its bioactive compounds, tea consumption is considered to have many potential health benefits. Although generally regarded as a safe beverage, some of its constituents may be associated with adverse effects, particularly at high doses or in concentrated extracts. Tea polyphenols (catechins and tannins) can form insoluble complexes with non-heme iron, reducing its absorption and bioavailability. Catechins may affect the activity of cytochrome P450 (CYP) enzymes involved in the metabolism of many commonly used prescription drugs. For instance, EGCG showed competitive inhibition of CYP1A2, CYP2B6 and CYP2C8, and non-competitive inhibition of CYP3A [26,27,28]. These types of interactions can potentially lead to a lack of therapeutic effects and/or enhanced side effects of the drugs used, due to their prolonged processing. Furthermore, tannins and polyphenols may also inhibit digestive enzymes, causing gastrointestinal disturbances [1,28,29]. High doses of catechins in turn may be associated with risk of hepatotoxicity. Animal studies (on mice and rats) demonstrated histopathological changes in liver (hepatocyte necrosis, hyperplasia of oval cells and bile duct) and significant depletion of glycogen, following administration of high concentrations of different tea extracts for several weeks. Mortality due to liver necrosis was also observed, especially with increasing doses of tea extracts and prolonged time of their administration [30,31]. Rare cases of acute liver injury were also described in humans (mainly in children) after intake of excessive amounts of tea [1]. Types of tea with a high content of caffeine can cause several related adverse effects, including increased blood pressure and heartbeat (which may put sensitive individuals at risk), insomnia and anxiety. High concentration of caffeine can disrupt calcium metabolism, which could result in its deficiency and therefore an increased risk of osteoporosis. Finally, with regular consumption, one can develop tolerance and dependance on caffeine, with potential withdrawal symptoms (headache, fatigue, impaired concentration, lowered mood) when it is not provided [32]. In addition, tea extracts might also be contaminated with heavy metals (lead, aluminum), with their concentrations increasing to the levels considered as unsafe, during longer steeping or brewing [33]. For safety reasons, moderate intake of tea, avoiding types with high concentrations of catechins and caffeine and avoiding consumption on an empty stomach, should be recommended to children and pregnant or breastfeeding women; all individuals should exercise caution with regard to health conditions and the use of medications and supplements [1].

### 3.2. Effects of Tea Compounds upon CNS

**Figure 2:**
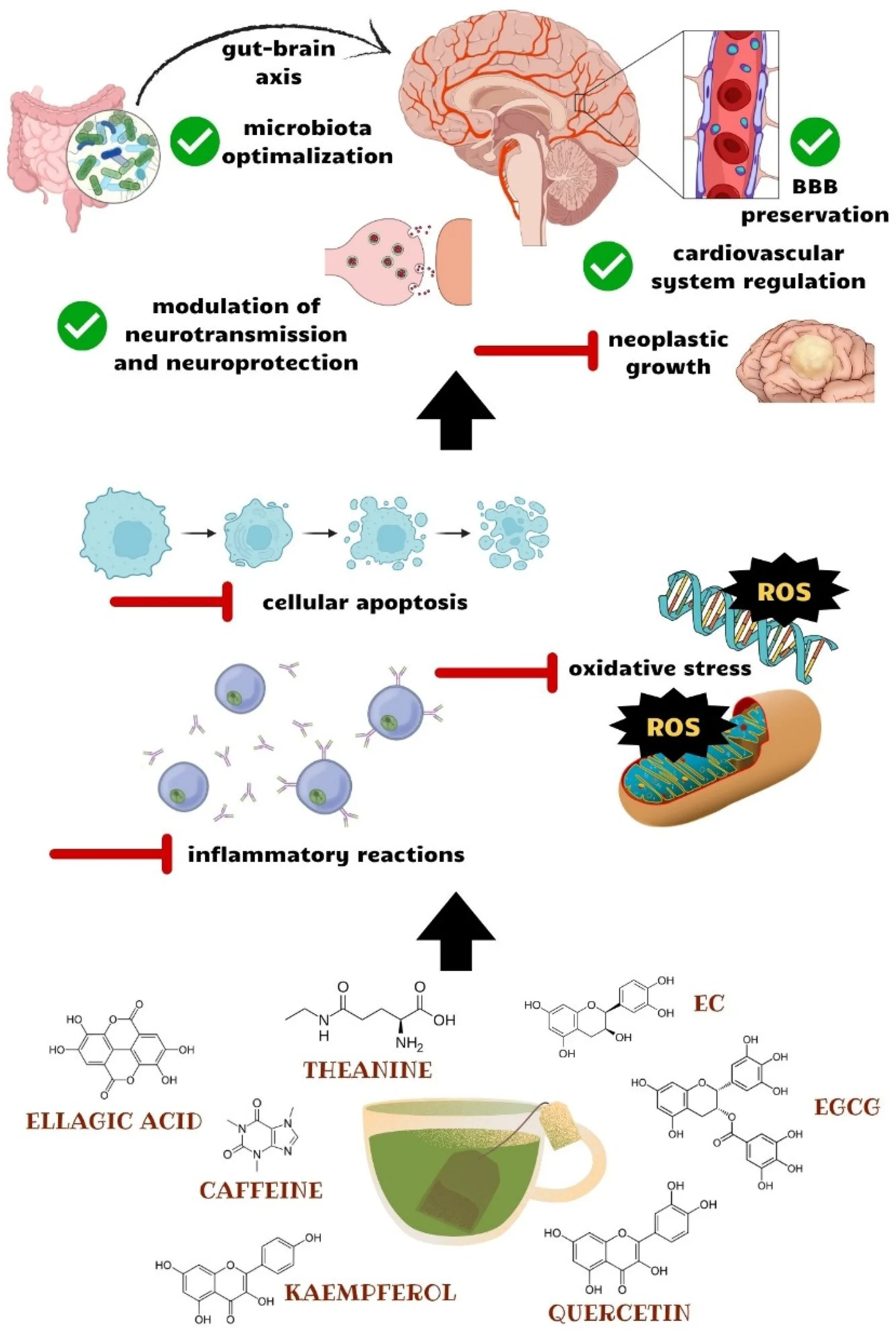
Potential mechanisms of the tea compounds impact upon CNS. Abbreviations: BBB—blood–brain barrier; EC—epicatechin; EGCG—epigallocatechin gallate; ROS—reactive oxygen species. 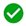
—support/activation 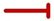
—suppression.

Multiple properties of tea compounds, associated with cellular defense mechanisms, can be particularly beneficial for the nervous tissue (Figure 2). Predominant antioxidant activity of tea polyphenols allows the prevention of neuronal injury, associated with harmful effects of free radicals (ROS, RNS): destruction of proteins involved in cellular structures and toxic products of lipid peroxidation. Due to high energy demands of neurons, inhibitory effect of catechins (mainly EGCG) on Ca^2+^ overload-related mitochondrial dysfunction and imbalance between their fusion and fission cycles also plays an important role in preservation of neuronal energetic stability [34,35].

Chelating characteristics of catechins prevent deposition of iron in neurons and microglia (with involvement of ferroprotein 1 and divalent metal transporter 1), which is another relevant mechanism contributing to neurodegeneration. Other neuroprotective mechanisms of tea polyphenols include regulation of autophagy and endocytosis (towards elimination of neurotoxic substances) and reduction in neural apoptosis (via suppressed activation of caspases, mitochondrial dysfunction and calcium overload). Furthermore, catechins act as antagonists of dual specificity tyrosine phosphorylation-regulated kinase (DYRK1A), which substantially contributes to impaired neurogenesis, neural differentiation and plasticity, and ultimately to cell death [35,36]. In turn, inflammatory injury to nervous tissue can be effectively prevented and modulated by catechins and flavonoids through Toll-like receptor 4 (TLR4)/NF-κB signaling pathway, which affects function of immunocompetent cells and microglia phenotypes, as well as through suppression of pro-inflammatory cytokines (e.g., TNF-α, IL-1β) and COX-2 expression [34].

Antioxidant and anti-inflammatory properties of tea polyphenols are associated with their relevant supportive effects on the blood–brain barrier (BBB), that restricts the passage of molecules and maintains the homeostatic balance between brain parenchyma and extracellular fluid [37,38]. Polyphenols contribute to preservation and stabilization of endothelial function through multiple mechanisms: inhibition of pro-inflammatory cytokine expression; modulation of the Nrf2/HO-1 pathway to reduce iNOS-mediated NO production and ROS levels; and activation of phosphoinositide 3-kinase (PI3K)/protein kinase B (Akt)/endothelial nitric oxide synthase (eNOS) signaling pathway. Furthermore, tea compounds were shown to regulate angiogenesis processes (modulating expression of vascular endothelial growth factor—VEGF and inhibiting Notch1 signaling pathway), promoting BBB formation and its appropriate function [7,39]. Active compounds of tea (and their metabolites) demonstrate distinct permeabilities across the BBB, depending on their chemical characteristics and the underlying transport mechanisms. In vitro BBB models showed that catechins demonstrate limited BBB permeability (more efficient for EC and gallic acid than for EGCG and EGC), which can be enhanced by their molecular configuration, interactions with transporters and lipophilicity. These observations can be relevant for optimal delivery strategies, which would allow better penetration of tea compounds into CNS and exertion of their beneficial properties [40,41,42] (Figure 2).

Another aspect of potential impact of tea compounds upon CNS is associated with gut–brain axis (GBA)—bi-directional communication network between intestine and CNS, encompassing metabolic, endocrine and immune interactions, linked with autonomic nervous system and hypothalamic–pituitary–adrenal (HPA) axis. In particular, content and function of gut microbiota have been demonstrated to affect significantly maturation and differentiation of neurons and glial cells, as well as functional integrity of the BBB. Thus, increasing attention has been paid to the role of gut microbiota in the background for CNS disorders [43,44]. Tea polyphenols and polysaccharides, during their ingestion and processing in the gastrointestinal tract, interact bidirectionally with gut microbiota, modulating its composition and abundance. They cause an increase in the number of beneficial bacterial strains (e.g., *Bifidobacterium*, *Lactobacillus*, *Akkermansia*, *Lachnospiraceae*, *Prevotellaceae* and *Ruminococcaceae*), while suppressing pathogenic ones (e.g., *Enterococcus*, *Shigella flexneri*, *Escherichia coli*, *Desulfovibrionaceae* and *Helicobacter*). Favorable effects of such modified microbiota profile include: correcting the metabolic imbalance of short-chain fatty acids (i.a. increasing production of butyrate), enhancing digestive enzyme activities and tightening of intestinal barrier, as well as downregulation of pro-inflammatory mechanisms and preventing production of neurotoxic metabolites, which are supposed to contribute to harmful impact of gut dysbiosis on CNS. Moreover, the gut microbiota is responsible for the biotransformation of polyphenols into their active metabolites, relevant for the synthesis of neurotransmitters [6,44,45,46] (Figure 2).

Particular tea compounds may either directly exhibit neuroactive properties (in their basic form or after being converted to active metabolites), or modulate function of other neurotransmitters. Gamma-aminobutyric acid (GABA) and kynurenic acid were detected in all types of tea, with the highest concentration in white tea and moderate concentrations in green and black tea (as a result of particular processing stages—withering, fermentation, etc.). Small amounts of dopamine have also been found mainly in oolong and green tea [47]. Catechins and flavonoids interact with multiple neurotransmitter systems in CNS (Table 2). Catechins (especially EGCG) were shown to enhance dopaminergic and cholinergic transmission, but also to mediate synaptic inhibition, interacting with GABA-A and glycine receptors [34,48,49]. Caffeine demonstrates mainly excitatory effects: acting primarily through adenosine receptor antagonism, it increases catecholamines and acetylcholine release, facilitates glutaminergic transmission and suppresses effects of GABA [50,51]. In turn, L-theanine, due to its structure, mainly competes as co-agonist for NMDA receptors, as well as increases the level of GABA, but also promotes dopamine release and increased concentration of serotonin [52] (Table 2). For some of these compounds, their effects on particular neurotransmitters appeared to be dose-dependent or limited to selected areas of the brain. Furthermore, it should be considered that the final effects of tea consumption result from interactions between its constituents [53].

These interactions with neurotransmitters contribute to the impact of tea compounds upon specific physiological functions of CNS. Catechins are considered to optimize neurotransmitter levels, supporting long-term synaptic adaptation and neuroplasticity. Thus, they may positively affect cognitive processes (especially memory and learning) [35]. Caffeine acts as a stimulant, enhancing alertness and vigilance and reducing fatigue. It was shown to increase level of attention and shorten reaction time, optimizing cognitive performance and modulating its circadian rhythm. Furthermore, it may boost both motor activity and mood, enhancing function of reward system [50]. L-theanine exhibits calming and relaxing effects: it can lower markers of stress via regulation of HPA axis and is regarded as a potent sleep modulator (improving sleep efficiency and self-reported sleep satisfaction, reducing nocturnal awakenings). However, there is also evidence that L-theanine can promote increased resting-state and event-related activity of neuronal networks. In electroencephalographic studies, L-theanine was shown to enhance alpha wave activity at rest and increase its synchronization during performance of cognitive-engaging task. These findings suggest its potential to induce a state of relaxed alertness, with more efficient attentional processing. The results of investigations of L-theanine’s pro-cognitive effects are ambiguous, with some improvement after L-theanine intake confirmed only for visual information processing and reaction time [54]. Unique combination of these bioactive compounds in a tea beverage may optimize their impact on CNS function. The study using functional MRI demonstrated that concomitant intake of caffeine and L-theanine in healthy persons modulated activation in the executive control and alerting/orienting cortical networks and improved focused attention during a demanding cognitive task. Complementary effects of caffeine, L-theanine and catechins are also supposed to provide an appropriate balance between alertness, arousal and a calming state [39,50,55].

## 4. Effects of Tea and Its Compounds in Particular CNS Diseases

### 4.1. Alzheimer’s Disease and Other Types of Dementia

Dementia is a broad term for condition associated with progressive decline in cognitive and behavioral functions to a degree that interferes with daily activities. Dementia constitutes a major cause of disability, dependency and mortality among older people, and has become substantial clinical and social concern, with ca 55 million cases worldwide and an incidence of more than 10 million new cases per year [42].

Alzheimer’s disease (AD), a neurodegenerative disorder, represents the most common cause of dementia (60–80% of cases) [56]. The pathological background of AD is characterized by the extracellular buildup of β-amyloid (Aβ) plaques and the intracellular aggregation of neurofibrillary tangles (NFTs), which consist of hyperphosphorylated tau protein. Abnormal protein aggregates directly cause neuronal injury (i.a. disrupting cytoskeleton and axonal transport and causing mitochondrial dysfunction), as well as initiate a cascade of events, including microglial activation, release of inflammatory cytokines and reactive astrocytosis. These processes lead to a diffuse loss of synapses and neurons (mainly cholinergic ones), predominantly within the limbic system (e.g., hippocampus) and the cerebral cortex [57,58]. As a result, patients suffering from AD experience progressive cognitive decline in multiple domains (particularly memory and visuospatial skills impairment), which ultimately makes them unable to function independently in society [56].

Other frequent causes of dementia include neurodegenerative disorders (frontotemporal dementia—FTD and Lewy body dementia—LBD) and cerebrovascular disease (vascular dementia—VD). Pathology underlying FTD is associated with aggregation of misfolded proteins in neurons and glial cells, involving tau, the transactive response DNA binding protein of 43 kDa (TDP-43), and fused in sarcoma (FUS) protein. These aggregates result in atrophy and gliosis within frontal and temporal lobes. Progressive behavioral disturbances (impaired executive functions and control of social behavior) and deterioration of speech/language skills predominate in clinical manifestation of disease [59,60].

The main pathological hallmark of LBD is the cytoplasmic deposition of misfolded α-synuclein protein in neurons of cortex, limbic system and basal ganglia. Clinical presentation comprises cognitive decline (affecting domains of attention, visuospatial skills and executive functions), psychiatric symptoms (visual hallucinations and delusions), accompanied by movement disorders (features of parkinsonism—see Section 4.2) and sleep disturbances [61]. VD develops in the course of cerebrovascular disease (following a stroke in the area important for cognitive functions or multiple ischemic infarcts as a result of small vessel disease—see Section 4.5). It is usually characterized by a stepwise decline and a fluctuating course of cognitive deficits in several domains, accompanied by other signs of motor, sensory or autonomic dysfunction, depending on localization of ischemic lesions [62].

Currently used therapies for dementive disorders focus on relieving the symptoms and modulating neurotransmission, while treatment targeted at the underlying disease mechanisms remains hardly available [56,58,63,64]. Parallel to extensive investigation into the search for new therapeutic options, the influence of lifestyle factors on the risk and progression of dementia is also being explored. Among these factors (i.a. physical activity, smoking, alcohol consumption and diet) [63,64], food components with potential neuroprotective properties have gained attention, including compounds of tea [56].

#### 4.1.1. Experimental Studies

Due to their antioxidant, anti-inflammatory and anti-apoptotic properties, tea catechins (especially the most abundant EGCG), have been investigated for their neuroprotective effects. EGCG was shown to exhibit an antioxidative effect in vitro in neuronal primary cultures, where it inhibited the toxic effects of 3-hydroxykynurenine, resulting in attenuation of oxidative stress, caspase activity and cell apoptosis [65]. In several experimental models of dementive disorders, administration of catechins effectively suppressed aggregation of pathologic proteins. Rezai-Zadeh et al. reported that EGCG reduced Aβ generation both in murine neuron-like cells (N2a) transfected with the human Swedish mutant amyloid precursor protein (APP) and in primary neurons derived from Swedish mutant APP-overexpressing mice [66]. In another experiment by these authors, using the APPSw transgenic mouse model, both intraperitoneal and oral treatment with EGCG suppressed the phosphorylated tau isoforms and regulated tau protein profile [67]. The study evaluating effects of EGCG oral administration in the TgCRND8 and APP/PS1 transgenic mouse models (which express multiple APP mutations) demonstrated beneficial effects of treatment on aggregation of amyloid and tau proteins, as well as on cognitive performance (spatial memory and learning), especially in combination with treadmill exercise [68]. EGC and ECG were also shown to cross the blood–brain barrier and mitigate the toxicity of Aβ oligomers, reducing their aggregation in plaques and preventing neuronal injury in the brains of APP/PS1 mice [69].

Furthermore, in vitro studies revealed that EGCG applied to the cells with mutant form of TDP-43 constrained pathological degradation of this protein and promoted formation of its non-toxic aggregates [70]. In another cellular model, catechins were also demonstrated (with the use of electron microscopy and biochemical analysis) to suppress tau hyperphosphorylation and inhibit aberrant tau filament formation, mainly through formation of soluble tau aggregates but without affecting tau interaction with microtubules [71].

Beneficial effects of catechins on cognitive performance in animal models of dementia were also associated with diminished indices of oxidative stress. In a streptozotocin-induced dementia rat model, short-term oral administration of EGCG reversed cognitive deficit and caused significant reduction in ROS and NO levels [72]. Similarly, oral intake of green tea catechins solution for several weeks prevented amyloid-induced cognitive impairment in rats and resulted in decreased levels of lipid peroxide and ROS in plasma and hippocampal specimens [73].

Kaempferol (KPF), another tea-derived polyphenol, also appears to possess antioxidant and neuroprotective properties, as well as ability to modulate neurotransmission [74]. In vitro study by Hanaki et al. showed that KPF could prevent 42-mer β-amyloid protein (Aβ42) aggregation, but without autooxidation (as in case of catechins). KFP was supposed to inhibit elongation rather than nucleation phase of this process and interact mainly with small aggregates of Aβ42 [75]. Neuroprotective and anti-inflammatory potential of KFP was further explored in glutamate-treated hippocampal neuronal cells and lipopolysaccharide-stimulated microglial BV2 cells. Proposed mechanisms of KFP effects included: regulation of the expression levels of proteins, such as Bcl-2, Bid, apoptosis-inducing factor, and MAPK [76], as well as downregulation of pro-inflammatory TLR4, NF-κB, JNK and AKT pathways [77]. Antioxidant and anti-inflammatory KPF properties were confirmed on ovariectomized rat model of streptozotocin-induced dementia [78]. KFP, administered intraperitoneally, significantly improved spatial learning and memory. As the background for these effects, significant increases in the levels of superoxide dismutase and glutathione, along with decreases in TNF-α and malondialdehyde levels were observed in the brain tissue [76,77,78]. When administered to transgenic *Drosophila* expressing human Aβ42 protein, KPF significantly delayed loss of their motor and behavioral abilities, acting through the inhibition of acetylcholinesterase activity and oxidative stress (revealed in their head homogenates) [79]. Modulation of cholinergic transmission by KPF was also demonstrated in the study by Zarei et al.: intracerebroventricular micro-injections of KPF improved learning and memory tasks performance in Wistar rats with scopolamine-induced cognitive impairment, also in combination with other non-selective and selective acetylcholine antagonists [80].

Polyphenolic acids contained in tea also exhibit neuroprotective properties, mediated by a reduction in oxidative stress and synaptic dysfunction [81,82]. Experiments on aluminum-chloride (AlCl_3_)-induced AD in adult Wistar rats showed that nutritional supplementation of gallic acid ameliorated learning and memory deficits, restored the decrease in antioxidant enzymes and levels of most neurotransmitters (except norepinephrine). In addition, it also prevented formation of neurofibrillary tangles and Aβ plaques in hippocampal external granular layer, suggesting a protective effect with regard to neurotoxic activity of AlCl3 [83]. Ellagic acid, administered intraperitoneally (especially in higher doses) to Wistar rats, significantly reversed memory deficits caused by scopolamine-induced cognitive impairment and improved behavioral parameters [81,82], without alterations in locomotion skills [84].

Positive impact of tea polyphenols upon cholinergic transmission was consistently displayed in other experimental studies. Regular feeding with diet enriched in tea polyphenols, prior to initiation of scopolamine-induced cognitive impairment in mice, significantly reversed related memory and behavioral deficits, as well as markedly inhibited acetylcholinesterase activity in the animals’ brains [85]. Oral administration of green tea extract to young and old Wistar rats allowed improvement in learning and memory task performance, which was associated with decline in acetylcholinesterase activity, but only in the older animals, suggesting a protective effect for age-related cognitive deficits [83,86].

Apart from polyphenols, another bioactive tea compound—L-Theanine (LTA)—has also been shown to counteract neurodegenerative processes in animal models of dementia. In the study by Elbaz et al., LTA administered intraperitoneally to mice with scopolamine-induced amnesia alleviated memory and learning deficits and reduced the number of Aβ plaques in hippocampus and cortex. LTA stimulated AMP-activated protein kinase (AMPK), microtubule-associated protein-1 light chain-3II (LC3II) and beclin 1, while decreasing phosphorylated levels of protein kinase B (p-AKT) and mTOR. Increased levels of the brain-derived neurotrophic factor (BDNF) and glutathione, and lowered levels of caspase-3, TNF-α and malondialdehyde were also noted. In a comparison between reference groups, the effects of LTA were similar to donepezil (acetylcholinesterase inhibitor) but were abolished by administration of chloroquine (autophagy blocker). These results indicate that the boosting of autophagy and reduction in oxidative stress are the main mechanisms by which LTA of promotes neuronal survival [87,88]. In another study on APP/PS1 mice, oral administration of LTA for several days improved memory task performance. Furthermore, in electrophysiological experiments using murine hippocampal slices, bath application of LTA facilitated hippocampal synaptic transmission and improved long-term potentiation through activation of dopamine D1/5 receptor and protein kinase A (PKA) pathway. These effects were observed only in APP/PS1 mice but not in the wild-type mice, while LTA elevated hippocampal levels of dopamine and noradrenaline in both groups [87].

#### 4.1.2. Epidemiological and Interventional Studies in Humans

Considering potential neuroprotective effects of tea and its compounds, vast epidemiological analyses were undertaken to evaluate their role in prevention of cognitive decline associated with aging and dementive disorders.

Meta-analysis of 26 observational studies (containing 52,500 participants from Asia, Europe, America and Australia) showed that daily tea consumption was inversely associated with the risk of overall cognitive decline and mild cognitive impairment in the elderly. However, no such association was demonstrated specifically for AD [89]. Another pooled analysis of seven prospective cohort studies, covering ca 410,000 individuals, revealed a significant relationship between consumption of green or black tea and reduced risk of all-cause dementia, as well as a risk of VD and (with lower significance) AD considered separately. Furthermore, subgroup analysis showed that tea intake could reduce the risk of dementia to a greater degree among elderly, smokers, apolipoprotein (*APOE*) ε4 carriers and those declaring less physical activity [90]. Vast evidence for protective potential of tea was provided by findings from UK Biobank, prospective cohort study comprising more than 300,000 participants during ca 10-year follow-up. After adjustment for demographics, lifestyle and dietary factors and *APOE* ε4 status, those with moderate consumption of tea (1–6 cups per day) were by 16% less likely to develop dementia in comparison with non-consumers. Lowering overall risk of dementia was greater in males and middle-aged ones, and it was greater for VD than AD [91]. Similar relationship was demonstrated for consumption of caffeine (in different beverages, including tea), with intake > 100 mg/day associated with lower risk of all-cause dementia and AD [92].

For the subgroup of ca 38,000 participants of UK Biobank with neuroimaging data available, additional analysis was conducted to examine the relationships between tea intake, risk of dementia and measures of brain atrophy. Non-linear correlation was confirmed between moderate tea intake (4–5 cups per day) and lower risk of dementia incidence, although no association was found directly with rate of cognitive decline. Moderate consumption of tea was also positively associated with regional gray matter volumes (including anterior parahippocampal gyrus, cuneal cortex, and the frontal lobe) and negatively associated with volumes of anterior cingulate cortex and white matter hyperintensities. It was suggested that neuroprotective potential of tea consumption could be linked with mitigating brain atrophy and impeding development of dementia [93].

With regard to caffeine intake, the studies evaluating its association with risk of dementia presented ambiguous results. The meta-analysis (comprising 20 observational studies with overall 31,479 subjects), focused on association between caffeine intake (in coffee or tea) and risk of cognitive disorders, failed to demonstrate significant relationships. In subgroup analyses, five studies were separately listed, in which tea consumption was not specifically associated with the risk of AD [94]. According to the data from the Rush Memory and Aging Project (888 participants aged >60 years), caffeine intake (in different beverages) did not show significant relationships with incidence of dementia or cognitive decline, but it was inversely associated with postmortem presence of Lewy bodies in brain tissue samples (for intake > 100 mg/day specifically with their neocortical type). However, no such associations were found with other pathological findings [92]. In a case–control report by Maia et al., daily caffeine intake over the preceding 20 years was retrospectively compared between people diagnosed with AD and healthy elderly subjects and turned out to be significantly lower in the AD group. Moreover, these results were independent of other possible confounding variables (e. g. hypertension, diabetes, smoking, alcohol consumption, gastric disorders, heart disease, and family history of dementia) [95]. In a cohort study involving elderly subjects (≥65-years), Santos et al. observed negative association between daily caffeine intake within a year preceding baseline assessment and risk of cognitive decline (decrease in Mini Mental State Examination/MMSE/score) within 3 years of follow-up, but this association was significant only for women [96]. Similar sex-related differences were observed in population-based cohort study [97]: women with high rates of caffeine consumption showed less decline in verbal and visuospatial memory over 4 years (effects increasing with age), but no such relationship was found in men.

Based on the results of experimental findings, observational studies and randomized clinical trials were conducted to explore the role of tea polyphenols in prevention or amelioration of dementia (Table 3).

Observational trial held for 12 years on 1329 people aged ≥65 years showed positive association between polyphenol-rich diet (including regular tea consumption) and lower risk of dementia [98]. In Japanese pilot study (NCT01594086) on a small group of subjects with AD or VD, regular intake of green tea extract for 3 months resulted in improved performance on MMSE, especially regarding the short-term memory domain, and lowered level of serum triglycerides [100]. However, in another study by these authors, daily administration of powdered green tea extract for 12 months to the subjects with cognitive impairment did not result in significant improvement in MMSE performance, compared to placebo. Intake of green tea catechins lowered the levels of malondialdehyde-modified low-density lipoprotein, which suggested prevention of oxidative stress [101]. In randomized placebo-controlled trial [102], administration of extract combining catechins and LTA for 16 weeks to the subjects with mild cognitive impairment (MCI) improved memory, selective attention and reading skills (assessed by a battery of cognitive tests) in comparison with placebo. Electroencephalogram performed in selected subjects after a single dose of catechins/LTA extract, revealed increased bioelectrical activity during opening eyes and reading [102]. The PENSA study [99] explored combined effects of oral EGCG (or placebo) and multimodal lifestyle intervention in elderly APOE-ε4 carriers with subjective cognitive decline. Improved cognitive performance (assessed by a battery of tests) was noted after 12 months in those treated with EGCG in comparison with placebo, approaching statistical significance.

Further clinical trials investigating effects of EGCG or caffeine upon cognitive decline in the patients with AD have been completed but have not yet yielded published results, or are still ongoing (Table 3).

### 4.2. Parkinson’s Disease

Parkinson’s disease (PD) is another common neurodegenerative disease, affecting mostly elderly people [103]. Main pathologic hallmark of PD is degeneration of dopaminergic neurons in substantia nigra in midbrain, accompanied by the presence of eosinophilic inclusions (Lewy bodies), which consist of misfolded α-synuclein. Background to this neurodegenerative process is complex and multifactorial, with neuroinflammation and oxidative stress playing pivotal roles. Activation of microglia, with release of pro-inflammatory factors (e.g., TNFα, IL-1β, NO) and stimulation of protein kinase R (PKR), seems reciprocally associated with formation of misfolded α-synuclein aggregates, disturbances of their degradation and subsequent neuronal injury. Furthermore, α-synuclein aggregates in Lewy bodies contribute to dysfunction of mitochondrial membrane, as well as release of free radicals and NO synthesis, resulting in oxidative stress development. Due to insufficient antioxidant capacity (e.g., low glutathione level), together with metal ion accumulation, nigrostriatal neurons become extremely prone to toxic injury related to impaired dopamine metabolism (increased MAO-B activity, excessive amount of H_2_O_2_, endogenous dopamine oxidation). All these processes lead to neuronal death and impairment of dopaminergic transmission within basal ganglia [104,105,106].

Typical clinical symptoms of PD include slowness and reduced amplitude of movement (bradykinesia), increased muscle tone (rigidity) and rest tremor. They are accompanied by non-motor symptoms, such as cognitive deterioration, depression, sleep disorders, and dysautonomia. Progressive course of disease, often associated with fluctuations of symptoms, significantly influences daily activities and quality of life in PD patients. Currently, no preventive or causal therapy is available for PD and therapeutic strategies are focused on restoring impaired dopaminergic transmission. Levodopa (a precursor of dopamine) is considered a gold standard of treatment; dopaminergic receptor agonists and inhibitors of dopamine degradation are also used. Deep brain stimulation with electrodes implanted into basal ganglia via stereotactic neurosurgery can be applied in selected patients in advanced stages of PD [107,108].

#### 4.2.1. Experimental Studies

Anti-inflammatory and antioxidant properties of tea-derived catechins and flavonols with their potential to prevent or inhibit damage to nigral dopaminergic neurons, have been explored in many experimental studies [109].

In vitro models showed that EGCG reduced microglial activation and neuronal impairment in SH-SY5Y and rat mesencephalic cultures. Simultaneously, EGCG decreased iNOS and TNF-α expression in lipopolysaccharide (LPS) activated microglia, which inhibited their pro-inflammatory activity [110]. In another study [111], SK-N-AS human neuroblastoma cells were exposed to 6-hydroxydopamine to develop in vitro PD model. Administration of catechin and EGCG to these cells increased their viability, displaying anti-inflammatory and anti-apoptotic effects (reduced immunoreactivity of caspase-3, TNF-α and IL-1β). Catechins applied to human HEK293T and SH-SY5Y cells suppressed the activity of metal ions and MAO-B and enabled detoxification of ROS and dopamine oxidation byproducts. They also modulated activity of nuclear factor erythroid 2-related factor 2—Kelch-like ECH-associated protein 1 (NRF2-Keap1) and PGC-1 pathways. As a consequence, reduction in endogenous dopamine oxidation-induced toxicity and emerging nigral neuron apoptosis were observed [112]. In an α-synuclein (h-αS) transgenic *Drosophila* model of PD, exposure to EGCG improved motor skills (climbing ability) of the flies. Molecular effects of EGCG included impact on voltage gated Ca channels with reduction in Ca^2+^ inflow into cells and their uptake by mitochondria, which prevented their dysfunction. Additionally, the attenuation of lipid peroxidation, oxidative stress and related apoptotic damage was observed [113]. Administration of EGCG via oral gavage to mice presenting 1-methyl-4-phenyl-1,2,3,6-tetrahydropyridine (MPTP)-induced model of PD, resulted in improved motor function and modification of peripheral immune response: decrease in pro-inflammatory cytokines (IL6 and TNFα) levels in serum, as well as increased CD3CD4 to CD3CD8 T-cell ratio. Furthermore, EGCG was found to protect tyrosine hydroxylase-positive cells in the substantia nigra from MPTP-induced neurotoxic damage [114].

The anti-inflammatory effects of EGCG-loaded liposomes were evaluated against LPS-induced BV-2 microglial cells and in the rat model of substantia nigra inflammation. In the in vitro assay, EGCG-loaded liposomes improved the LPS-induced production of BV-2 microglia-derived NO and TNF-α and scavenged a large proportion of free radicals. Intra-nigral injection of EGCG-loaded liposomes in rats attenuated production of LPS-induced pro-inflammatory cytokines and effectively restored motor impairment [115].

In another mouse model of PD (induced with α-synuclein preformed fibers—α-syn-PFFs), intraperitoneal injections of EGCG mitigated dyskinesia and anxiety. Effects of EGCG on immune response were demonstrated: it inhibited expression of pro-inflammatory cytokines (IL-6, IL-1, TNF-α) and enhanced the expression of anti-inflammatory ones (TGF-β, IL-10, IL-4). At the same time, there was reduction in accumulation of α-syn-PFFs in Lewy bodies, which prevented neurodegeneration [116]. In rotidone (ROT)—induced rat model of PD, intraperitoneal injection of EGCG prior to ROT administration prevented major part of the motor impairment and caused increase in catecholamines levels in the striatum. Antioxidative effects of EGCG included reduction in NO and lipid peroxidation levels, with increased activity of succinate dehydrogenase and ATPase, as well as protection of mitochondria. Furthermore, levels of neuroinflammatory and apoptotic markers were also diminished [117]. Antioxidative effects of EGCG were shown in another MPTP-induced PD mouse model. EGCG administered orally concomitantly with MPTP improved animals’ performance in motor behavioral tests, raised striatal dopamine concentration and expression of iron-export protein ferroportin in substantia nigra and diminished levels of protein carbonyls. Thus, EGCG mitigated MPTP-induced neurotoxicity [118].

Similar neuroprotective effects were demonstrated for experimental PD models for kaempferol (KPF). KPF improved striatal neuron injury induced by LPS in mice, by increasing the levels of tyrosine hydroxylase and postsynaptic density protein 95. In addition, KPF inhibited the production of pro-inflammatory cytokines (IL-1β, IL-6, TNF-α), reduced the level of monocyte chemotactic protein-1, and COX-2 and suppressed high mobility group box 1/Toll-like receptor (HMGB1/TLR) inflammatory pathway, thus protecting BBB integrity [119]. In another murine model of LPS- and α-synuclein -induced neurodegeneration, KPF reduced cleaved CASP1 expression, promoted autophagy in microglia (with involvement of ubiquitination) and reduced nucleotide-binding domain, leucine-rich repeat containing (NLRP3) protein expression, which in turn deactivated related inflammasome [120]. Exposure to KPF in mice with MPTP-induced model of PD resulted in the rise in striatal dopamine concentration, as well as motor function improvement. Moreover, KPF increased levels of natural antioxidants (SOD and glutathione peroxidase, GSH-PX) and reduced the level of malondialdehyde (MDA), mitigating oxidative stress [121].

#### 4.2.2. Epidemiological and Clinical Studies in Humans

Several epidemiological studies provided evidence for beneficial effect of tea consumption on the risk of PD development.

A prospective study of over 120,000 participants explored association between intake of total flavonoids from major food sources (including tea) and risk of PD. Male participants in the highest quintile of total flavonoids intake had a 40% lower risk of PD than those in the lowest quintile, after adjusting for several confounders. However, no such relationship was confirmed for women [122]. In the cohort study conducted in over 70,000 Chinese women, the risk of PD appeared to be inversely associated with drinking tea and passive tobacco smoking from their spouse, although none of these relationships reached statistical significance [123]. Moreover, a meta-analysis of 15 studies on caffeinated beverage intake (eight studies specifically concerning tea) with over 80,000 participants, proved a linear dose-dependent inverse relationship between tea and caffeine consumption and the overall risk of PD, which was stronger for men than for women [124].

The case-controlled Iranian study investigated non-genetic factors predisposing individuals for PD. Comparative analysis of 75 PD subjects and 75 controls demonstrated higher regular green tea consumption in the latter; with every extra glass of tea per day decreasing the risk of PD by 0.8 times [125]. Similar Japanese study involved 249 PD cases and 368 controls. After adjustment for demographic factors and other dietary habits, intake of coffee, black tea or Japanese and Chinese teas was significantly inversely associated with the risk of PD. In particular, inverse dose–response relationship between total caffeine intake and PD risk was observed [126]. Furthermore, the cohort Chinese study (comprising 4093 participants) explored the potential role of green tea consumption in slowing down the progression of PD, using two-sample Mendelian randomization analysis. A significant association was revealed between higher green tea intake and lower risk of non-motor PD symptoms occurrence, such as depression, dementia, insomnia and hyposmia, and their delayed onset in the course of disease (for depression and dementia). Moreover, weak relationship (of borderline significance) was found between drinking green tea and the progression of PD motor symptoms, assessed in Hoehn–Yahr scale. The results of this study suggested a potential preventive effect of green tea in PD, which might be applied in recommendations for lifestyle factor modifications for PD patients [127].

However, considering the promising results from experimental studies on particular tea compounds effects in PD, the amount of interventional clinical trials undertaken in this field has been surprisingly limited.

A randomized, placebo-controlled clinical study recruited ca. 400 untreated patients with early PD from several Chinese centers, to evaluate the safety, tolerability, and efficacy of green tea polyphenols in slowing disease progression. Green tea polyphenols vs. placebo, administered daily at different doses for 6 months (followed by 6 months of polyphenols administration to the whole study group) provided mild relief in PD symptoms and were well tolerated with slight increase in insomnia [128]. Other clinical trials focused on potential effects of caffeine, assuming its excitatory properties and positive impact on dopaminergic transmission. Phase 2 and 3 studies (NCT00459420) evaluated the effects of caffeine intake on daytime somnolence. Participants received increasing doses of caffeine (vs. placebo) for 6 weeks, in addition to regular anti-parkinsonian treatment. Caffeine intake resulted in a slight (border of significance) improvement of excessive daytime somnolence but also restored objective motor function measures and was well tolerated [129] (Table 4). Disappointing results emerged from another trial (NCT01738178) evaluating impact of caffeine on motor and non-motor PD symptoms. Caffeine capsules administered (vs. placebo) for 6–18 months not only failed to improve motor functions but also slightly increased occurrence of dyskinesia and cognitive deterioration, as well as caused transient enhancement of daily somnolence [130] (Table 4). A few similar trials were conducted in small groups of PD patients but despite their reported completion, their results are not available.

### 4.3. Huntington’s Disease

Huntington’s disease (HD) is a rare hereditary neurodegenerative disease with the onset of clinical presentation around 40–50 years of age and progressive course. The mode of inheritance is autosomal dominant, with trinucleotide CAG repeat expansion within the Huntingtin (HTT) gene on chromosome 4. Altered HTT gene translates into mutant huntingtin protein (mhtt) with elongated polyglutamine (polyQ) tract, prone to misfolding and forming fibril aggregates in striatal and cortical neurons. These toxic inclusions impair mitochondrial function, transcription, and axonal transport, leading to neuronal death. As the altered HTT gene is passed from one generation to the next, the size of the CAG repeat often increases and reaches the range (more than 36 repeats) determining clinical manifestation of disease. The core symptoms comprise involuntary movements affecting the whole body (chorea), accompanied by deterioration of cognitive functions, behavioral and affective disorders. In later stages of HD, the slowness of movements with apathy and dementia predominate, leading to severe disability and loss of independence. So far, there is no available cure or disease modifying therapy for HD. Agents decreasing dopamine levels (vesicular monoamine transporter type 2 inhibitors), antipsychotics and cholinesterase inhibitors are used to relieve the symptoms [131].

Potential neuroprotective properties of tea-derived polyphenols encouraged evaluation of their adverse impact on abnormal mhtt protein aggregation in neurons, i.a. through antioxidative effects and interactions with cellular lipid membranes [36].

#### Experimental Studies

On in vitro assays, EGCG was shown to interfere with early stage of mhtt aggregation and modulate its misfolding and oligomerization. Aggregation and cytotoxicity of polyQ-mediated mhtt protein were also significantly reduced by EGCG in the yeast model of HD. These effects were also demonstrated in vivo: EGCG fed to transgenic HD flies (overexpressing a pathogenic mhtt protein), allowed improvement in their motor function and photoreceptor degeneration [132]. Another study on in vitro HD model confirmed that EGCG can effectively suppress mhtt aggregation. In contrast to curcumin, EGCG inhibited mhtt fibril formation independently of the presence of lipid vesicles (1-palmitoyl-2-oleoyl-glycero-3-phosphocholine or total brain lipid extract). These findings highlight the role of lipid membranes as a key modifier of the polyphenols ability to counteract abnormal protein aggregation [133].

A transgenic *Drosophila* model of HD was used to investigate the effects of green tea infusion of htt-induced degeneration. Supplementation with green tea improved motor activity of flies, indicating better functional integrity of the nervous system, and suppressed photoreceptor degeneration (evaluated with pseudopupil assay) induced by mutant htt. Furthermore, moderately increased lifespan was observed in mutant *Drosophila* (but not in the wild-type flies), suggesting selective effect of green tea on mhtt toxicity, rather than its general positive impact on viability [134]. Another tea polyphenol, quercetin, was supplemented by oral gavage to 3-nitropropionic acid (3-NP) induced rat model of HD. Quercetin administration improved rats’ performance in motor behavioral tests, as well as reduced or prevented damage to neurons and astrogliosis in striatum revealed in histopathological analysis. On the cellular level, quercetin supplementation reversed inhibition of respiratory chain complexes, restored ATP levels, attenuated lipid peroxidation and prevented mitochondrial swelling. Antioxidative effects also included restored activity of SOD and catalase along with thiol content [135].

Unfortunately, these promising effects have not been confirmed in clinical or observational studies in humans, despite a few attempts being undertaken. Randomized, placebo-controlled prospective multicenter trial (The ETON-Study) aimed at evaluation of EGCG administration for 12 months on motor functions, cognitive performance, neuropsychiatric symptoms and quality of life, as well as brain atrophy in HD patients. Although the trial is assigned as completed, no published results are available. The same status concerns the observational study (NCT03034122) evaluating the impact of caffeine consumption on progression of motor and cognitive dysfunction, as well as brain atrophy in patients with HD within 2 years [36] (Table 4).

### 4.4. Multiple Sclerosis

Multiple sclerosis (MS) is a chronic, polyphasic disease of the CNS, characterized by multifocal lesions throughout the brain and spinal cord. A complex background of MS involves two main components: immune-mediated inflammatory injury and degenerative process with axonal loss. Autoreactive immunocompetent T cells reach the CNS through disrupted BBB and initiate inflammatory cascade, targeted at myelin antigens. Inflammatory activity is mediated by cyto- and chemokines, as well as humoral response driven by B cells and activation of glial cells. Parallel to this process, there is a slowly expanding neurodegeneration, associated with ion channel dysfunction, oxidative stress and mitochondrial dysregulation, which ultimately leads to axonal loss. These components develop with different dynamics: flares of inflammatory activity predominate in the early phase of MS, later turning into diffuse and chronic inflammatory injury, while neurodegeneration is gradually escalating during advanced, progressive disease stages [136].

Because of its specific features (onset usually in young adults, long-lasting and hardly predictable course, accumulating disability due to variety of symptoms: motor and sensory disturbances, visual impairment, problems with gait and balance, bowel and bladder dysfunction), MS constitutes both clinical and social problem. Despite recent substantial progress in the field of MS diagnosis and treatment, there are still some challenges ahead. They include mainly management of the neurodegenerative component of MS background and prevention of progressive phases of the disease, but also amelioration of so-called “invisible disability” symptoms (cognition, fatigue, mental health issues) [137].

Anti-inflammatory and neuroprotective properties of tea compounds, targeted at both processes underlying MS background (and a cross-talk between them) made them an object of studies as potential therapeutic options in MS.

#### 4.4.1. Experimental Studies

Numerous experimental studies on animal models of CNS demyelination provided evidence for beneficial properties of tea-derived polyphenols.

Administration of EGCG to mice with experimental autoimmune encephalomyelitis (EAE) reduced symptoms of CNS inflammatory injury. Contributing mechanisms included: inhibition of pro-inflammatory signaling pathways (signal transducer and activator of transcription—STAT 1/3 and NF-κB), alteration of T cell subsets balance towards anti-inflammatory profile, as well as expression of adhesion molecules to regulate immunocompetent cells migration across the BBB [138,139]. In the study on another animal model, C57Bl/6 mice with cuprizone-induced demyelination were administered EGCG intraperitoneally. In comparison with the reference groups, their cerebral cortex specimens showed increased expression of proteolipid protein and oligodendrocyte transcription factor 1 [140]. Furthermore, EGCG combined with glatiramer acetate (immunomodulatory agent used in MS treatment) exerted neuroprotective effects, increasing protection from glutamate- and TNF-related apoptosis-inducing ligand (TRAIL)—induced neuronal cell death in vitro and promoting regeneration of hippocampal axons in an outgrowth assay [141]. Because such combined therapy applied in EAE mice showed partly contradictory effects of both agents used, other properties of EGCG were investigated. It was suggested that neuroprotection by EGCG in chronic EAE may involve impact on oxidative processes, including downregulation of heme oxygenase-1 HO-1 (compromised by its upregulation by glatiramer acetate) [142].

Ellagic acid (EA) and its metabolites delayed the onset and ameliorated symptoms of EAE and cuprizone-induced demyelination. The following effects of EA were revealed: reduction in pro-inflammatory cytokines (i.a. IFN γ), altered differentiation of Th1/Th17 cells, but also suppression of microglial and dendritic cells activity and prevention of oligodendrocytes apoptosis [143]. In addition, EA administered to EAE mice was demonstrated to remodel their gut microbiome composition with increasing abundance of bacterial strains (i.a. *Alloprevotella*, producing propionate). Inhibiting histone deacetylases activity and promoting acetylation by bacterial products further suppressed Th17 differentiation and release of inflammatory cytokines, alleviating EAE symptoms [144].

In turn, KPF applied to EAE mice reduced clinical symptoms of disease and exhibited multidimensional effects on CNS tissue: attenuated inflammatory injury mediated by IL-17 and CCL-19, counteracted neuronal ferroptosis (by reduction of iron overload and upregulation of ferroportin 1 expression) and promoted axonal remyelination (escalation in myelin basic protein content through activating the cAMP/CREB/ciliary neurotrophic factor (CNTF) axis [145].

#### 4.4.2. Clinical and Epidemiological Studies

Following these promising results of experimental studies, several clinical trials were conducted to investigate potential therapeutic effects of catechins and other polyphenols in MS (Table 5).

Platero et al. evaluated the impact of EGCG and coconut oil oral intake for 4 months upon the levels of IL-6, anxiety and functional disability in patients with MS. In the intervention group, compared to placebo, there was a decrease in levels of anxiety (measured by the State-Trait Anxiety Inventory) and disability (Expanded Disability Status scale score), while IL-6 concentration decreased in both groups. Combined effect of EGCG and ketone bodies from coconut oil was supposed to act through anti-inflammatory mediators and by modulating NMDA and GABA receptors [151]. Similar combination of EGCG and coconut oil, accompanied by isocaloric diet, was applied in the pilot study by Benlloch et al. [150], who investigated effects of such intervention on level of ketone bodies and anthropometric measures in MS subjects. After 4 months, increase in plasma beta-hydroxybutyrate, albumin and paraoxonase 1, as well as reduced waist-to-hip ratio and increased muscle percentage, were observed in the intervention group (in comparison with the controls), with putatively diminished cardiovascular risk.

However, the findings from other clinical trials with EGCG were rather disappointing. Phase I and II study aimed at evaluation of futility of Polyphenon E (green tea extract) oral intake as neuroprotective agent and its safety. In a small group of participants, EGCG failed to demonstrate neuroprotective properties (measured by N-acetyl-aspartate levels in magnetic resonance spectroscopy); furthermore, risk of hepatotoxicity was observed [146]. In another larger, multicenter phase II randomized controlled study, patients with relapsing-remitting MS received oral EGCG or placebo, in addition to stable disease-modifying treatment with glatiramer acetate, for 18 months. The treatment appeared to be safe and well tolerated but no superiority of EGCG (add-on to glatiramer acetate) to placebo was demonstrated for any of investigated outcomes: clinical (level of functional disability, relapse rate), radiological (presence of new and active lesions, rate of brain atrophy in MRI) or immunological (response to drug). In addition, EGCG plasma levels showed marked individual diversity [148]. Phase II randomized placebo-controlled trial recruited patients with progressive types of MS and aimed at assessment of oral EGCG impact upon rate of disability progression and measures of brain atrophy in MRI. After 36 months of observation, no group differences were revealed in clinical or radiological endpoints, although safety profile of EGCG was overall positive [149] (Table 5).

More relevant findings emerged from the clinical trial by Karegar et al. in which MS patients were randomly allocated to receive orally EA or placebo for 3 months. In the EA group, significant improvement was noted in the measures of anxiety, depression and fatigue. Moreover, supplementation of EA was associated with a reduction in serum levels of IFNγ, IL-17, NO and increase in IL-4, as well as decreased expression of Tbet and RORγt genes and increased—of GATA-3 gene. Influence of EA on differentiation of Th cells towards their anti-inflammatory profile, inhibition of microglial activation with subsequent modulation of hypothalamic–pituitary–adrenal axis were hypothesized as mechanisms responsible for observed clinical effects [152,153].

Due to increasing awareness of the role of environmental factors and their interactions with genetic predispositions in MS etiology, lifestyle factors (including dietary habits) have been investigated with regard to risk and severity of disease. Consumption of caffeine (contained in different beverages) was implemented in these analyses among other dietary factors, with divergent results. In the longitudinal assessment of the large cohort of white women (U.S. Nurses Health Study), no significant association was found between caffeine intake and the risk of MS [154]. Furthermore, genome-wide association study analyzing single-nucleotide polymorphisms related to habitual caffeine consumption in 375,833 participants, failed to establish a causal relationship between coffee intake and MS risk [155]. In large population-based case–control study, retrospective analysis showed reduced risk of MS in those who reported high consumption of coffee within 5–10 years prior to disease onset, but no such evidence was revealed for consumption of tea [156]. On the contrary, the case–control study evaluating lifestyle profile in people with first clinical manifestation of demyelinative disease (prior to establishing diagnosis of MS), demonstrated borderline significant trends for increased risk of such an event in those who had consumed more coffee but less tea in the preceding year [157]. The only study considering different types of tea, the case–control analysis with moderate sample size, revealed that consumption of black or green tea, as well as coffee, was associated with a reduced chance of developing MS [158].

In the study by Ivashynka et al., the impact of lifetime coffee and tea consumption was evaluated in relation to dynamics of disability accumulation (MS Severity Score). Although the findings did not reach statistical significance, the risk of falling into upper MSSS tertile appeared to be the opposite for coffee and tea, with the trend for lower risk for tea. Differences in composition of these beverages were discussed (lower amounts of caffeine and higher amounts of polyphenols in tea) and need for verification of these relationships in larger samples was highlighted [159].

### 4.5. Cerebrovascular Disease

Due to incidence of cardiovascular system disorders and consequences of vasogenic brain injury, cerebrovascular disease (CVD) constitutes nowadays one of the major health problems. Acute cerebral stroke is a leading cause of death and permanent disability worldwide. Chronic CVD in turn leads to progressive impairment of cognitive performance and mobility, affecting the patients’ daily functioning. Thus, prevention of CVD and its effective management are essential tasks challenging the healthcare system.

Insufficient blood supply to the brain (due to thrombosis, embolism, or systemic hypoperfusion) leads to cerebral ischemia, initially transient, but with prolonged duration and lack of compensation turning into irreversible necrosis. On a molecular level, energy failure initiates the release of excitatory neurotransmitters and oxidative stress, accompanied by induction of inflammation by activated microglia and astrocytes, which ultimately causes necrotic cell death. Disruption of BBB and subsequent cerebral edema further enhance ischemic damage to brain tissue. The current treatment of acute ischemic stroke is based mainly on interventions which cause reperfusion of cerebral tissue (including mechanical thrombectomy and i.v. thrombolytic therapy with recombinant tissue plasminogen activator—rTPA) However, their use is restricted by the limited time window and/or presence of risk factors for secondary bleeding. Furthermore, despite extensive search, effective neuroprotective agents, capable of saving the brain tissue from permanent ischemic injury, are still lacking. In view of antioxidative and anti-inflammatory properties of tea compounds, as well as their effects on vascular function, their use within dietary or pharmacological interventions is worth considering in prevention and management of CVD [160,161].

#### 4.5.1. Prevention of Stroke

There is strong evidence that intake of tea and its compounds may positively modify cardiovascular risk factors for CVD and thus play a role in its long-term prevention [162].

Hypertension and heart diseases are major risk factors for stroke and small vessel disease. Results of several meta-analyses showed that tea consumption tends to reduce the blood pressure (both systolic and diastolic), through such mechanisms as vasodilation from prostacyclin release, enhancement of NO production and oxidative stress reduction [163,164,165]. These effects on endothelial vasomotor dysfunction were also proposed as the background for protective role of tea polyphenols in patients with coronary artery disease [166]. In Japanese studies, regular consumption of green tea was found to be an independent predictor of coronary artery atherosclerotic stenosis, but mainly in men. Similarly, inverse association was revealed for green tea intake and risk of myocardial infarction [167,168].

Obesity and dyslipidaemia commonly predispose individuals to cardiovascular system diseases, including cerebral stroke. Effect of tea compounds on reduction of body weight may be exerted through decrease in intestinal absorption of lipids and proteins, consistently reducing calorie intake, as well as by stimulating AMP-activated protein kinase, which is bioavailable i.a. in adipose tissue, and supposedly also through inhibited ghrelin secretion with increased adiponectin levels [169,170]. In middle-age Japanese women, an inverse association was found between the green tea intake and high body mass index (BMI), body fat percentage and high cardio-ankle vascular index (CAVI) [169]. Another study comprising obese women demonstrated significant weight loss, reduced waist circumference, and a consistent decrease in total cholesterol (TC) and low-density lipoprotein (LDL) plasma levels after 12 weeks of supplementation with green tea extract [170]. Significant decrease in LDL level after EGCG supplementation (in beverage or capsule form) for 4–14 weeks was confirmed by the results of systematic review of 17 different studies on over 1300 people [171]. Moreover, a cohort study on over 80,100 healthy people showed that tea intake can slow age-related high-density lipoprotein (HDL) level decline, especially in men, aged ≥60 years, burdened with metabolic syndrome and a lower lifestyle score [172]. In some studies, green tea consumption was shown to reduce total cholesterol and oxidized low-density lipoprotein (LDL) cholesterol levels, parallel to lowering blood pressure [165,173].

In view of the above, potential benefits from tea compounds intake seem to be associated with their multidirectional impact on various risk factors for CVD. However, due to methodological differences and confounding factors, there are some inconsistencies in the results of these studies. In the clinical trial investigating the effects of epicatechin and quercetin supplementation for 4 weeks on multiple hemodynamic and metabolic indices, the only significant effects demonstrated for epicatechin included improved fasting plasma insulin and insulin resistance, while no relevant effects were noted for quercetin [174].

Nevertheless, the findings from prospective cohort studies confirm beneficial role of tea (especially green) in prevention of cardiovascular disease-related consequences. In large Japanese populations, followed for over 11 years, the risk of mortality from cardiovascular reasons (including CVD) was inversely associated with consumption of more than five cups of green tea per day, and this association was stronger than for other causes of death [175,176].

The evidence for preventive role of tea consumption has also been provided specifically with regard to cerebral stroke. Pooled data from nine studies, involving 4378 strokes in 194,965 individuals, showed that consumption of more than three cups of tea per day was associated with reduced (by 21%) risk of stroke occurrence and related mortality. The results were consistent for black and green tea and independent from the country of data origin [177]. Another meta-analysis of 14 studies, comprising over 513,800 participants with a median follow-up of 11.5 years, also found modest but significant association between increased daily tea consumption (up to three cups) and reduced risk of cerebral stroke (by 13%). These effects were more pronounced in women, drinkers of green tea and in Japanese studies. The associations remained significant after adjustment for potential confounding factors, including diabetes, hypertension, physical activity and dietary habits. It is worth highlighting that these relationships were relevant only for ischemic stroke, but not for intracerebral or subarachnoid hemorrhage [178].

#### 4.5.2. Protection from Brain Ischemic Injury

Potential neuroprotective effects of the tea compounds in acute ischemic injury of the nervous tissue (by attenuating inflammation, free radical accumulation and neurotoxicity of excitatory amino acids) were explored in several experimental studies [179].

Gundimeda et al. studied in vitro effects of polyphenols contained in green tea or their isolated components on PC12 neuroblastic cells (derived from the rat adrenal medulla), exposed to oxygen-glucose deprivation and then reoxygenation (OGD/R) [180]. Out of the isolated polyphenols, only EGCG and ECG effectively preconditioned PC12 cells against OGD-induced cell death. The putative mechanism was through stimulation of a 67 kDa laminin receptor, followed by induction of NADPH oxidase-dependent generation of ROS and ultimately—activation of isoenzyme protein kinase Cε, contributing to the cells’ survival [180]. Kim et al. investigated potentially protective effects of pre-treatment with EGCG in hypoxic model of BV2 murine microglia cells exposed to CoCl_2_ treatment. EGCG ameliorated increase in IL-6, iNOS and expression of COX-2, as well as suppressed hypoxia-inducible factor (HIF)-1α expression and generation of ROS associated with ischemic injury, via inhibition of NF-κB and activation of Nrf-2/HO-1 pathways. Furthermore, EGCG also suppressed apoptotic processes in hypoxic cells with cleavage of poly (ADP-ribose) polymerase (PARP) and caspase-3 [181].

In the animal model of brain ischemia (middle cerebral artery occlusion—MCAO) in C57BL/6 mice, followed by reperfusion after 28 days, effects of EGCG applied for several days into cerebral ventricular system were assessed in vivo and in vitro. Animals treated with EGCG presented with better outcomes of cerebral ischemia (improvement of neurologic deficit assessed in functional tests). Following EGCG treatment, neural progenitor cells isolated from animals’ subventricular zone at 14 days post-ischemia, showed enhanced proliferation and differentiation, with migration of neuroblasts and their functional recovery, supposedly due to neuroprotective response from M2 microglia phenotype and induction of AKT signaling pathway [182]. Another experimental study on similar model (MCO performed in rats) focused on effects of EGCG (injected before the procedure) during first 24 h of brain ischemia. Pretreatment with EGCG attenuated emerging neurological/behavioral deficits and increases in ROS and LPO levels. Analysis of isolated cerebral cortex specimens demonstrated several protective mechanisms of EGCG which supported cells viability: modulating interaction between apoptosis signal-regulating kinase 1 (ASK1) and thioredoxin, as well as thioredoxin expression in glutamate-exposed neurons, and suppressing increase in caspase-3 in response to glutamate exposure [183]. In the study by Lin et al., repeated intraperitoneal injection of EGCG for 10 days before induction of brain ischemia in rats (occlusion of common carotid and middle cerebral arteries) allowed the reversal of detrimental biochemical results of the injury: decrease in antioxidant enzymes (superoxide dismutase and catalase), increase in malondialdehyde, and disturbed proportion of trace elements (Mg, Zn, Se vs. Cu, Fe and Pb), demonstrated in analysis of cortex tissue supernatants [184]. You et al. in turn administered simultaneous treatment with rt-PA and EGCG to MCAO model in rats. Such combined intervention improved the neurobehavioral deficit and—in brain tissue samples—diminished extent of infarction, surrounding edema, and BBB disruption, even after application of treatment later than 3 h after ischemia onset. Proposed mechanisms of EGCG effect included ability to upregulate plasminogen activator inhibitor-1 and downregulate matrix metalloproteinases [185].

Similar effects in the rat model of focal brain ischemia were observed for another tea polyphenol—quercetin. Yao et al. demonstrated that quercetin improved neurological function and reduced volume of cerebral infarct after MCAO. At the tissue level, it allowed increased expression of BDNF, tropomyosin receptor kinase B (TrkB) and p-AKT, as well as suppressed cell apoptosis (with decrease in cleaved caspase-3), due to activation of corresponding signaling pathway [186]. Lee et al. found that quercetin administered after the induction of ischemia by photothrombosis, improved functional outcomes, reduced BBB permeability and brain edema and inhibited upregulation of matrix metalloproteinase-9 (MMP-9), without changing MMP-2 activity [187].

Some neuroprotective properties with regard to brain ischemia were proposed also for theanine. In transient forebrain ischemia induced by bilateral occlusion of the common carotid arteries in gerbils, intraventricular injection of theanine before this procedure prevented extent of neuronal death in hippocampal CA1 region in a dose-dependent manner [188]. Theanine administered intraperitoneally after MCA occlusion in mice helped to reduce the size of the cerebral infarct and alterations in the expression levels of neuronal nuclear protein (NeuN), glial fibrillary acidic protein (GFAP), and ionized calcium-binding adaptor molecule-1 (Iba 1), supposedly through activation of GABA receptors [189].

Despite such sound evidence from experimental studies, hardly any attempts have been made to replicate these findings in humans. Based on their previous study results [185], Wang & You [190] explored outcomes of ischemic stroke following the combined effects of EGCG oral administration and i.v. fibrinolytic treatment with rTPA in a unique clinical trial (involving 371 patients). For those patients in whom rt-PA was applied within 3–5 h of the onset of stroke symptoms, concomitant administration of EGCG (in comparison with placebo) was associated with greater reduction in neurological deficit (measured by means of NIHSS score) after 24 h. However, no such significant differences were observed in the group of patients treated with rTPA within a time window shorter than 3 h. Supplementation with EGCG (or placebo) was then continued for subsequent 7 days and clinical assessment of patients after this follow-up revealed similar findings: improvement in the stroke outcomes was greater in EGCG subgroup than in placebo subgroup, but only for the patients who had received fibrinolytic treatment within a longer window timeframe. In conclusion, the authors suggested that EGCG could effectively extend therapeutic window for fibrinolytic treatment and have a beneficial effect on short-term recovery after ischemic stroke. Because of correlations between NIHSS scores and plasma levels of matrix metalloproteinases (MMP-2 and 9), modulation of their activity (associated with disruption of BBB and neurotoxicity) was proposed as the main mechanism of EGCG action [190].

### 4.6. Brain Tumors (Gliomas)

Primary brain tumors, irrespective of their type, cause a serious burden to the lifespan and general condition of affected patients. Direct damage to cerebral tissue and compression of adjacent structures result in focal neurological deficit, as well as generalized symptoms of raised intracranial pressure. Histopathologically, the majority of primary brain tumors in adults are gliomas, among which glioblastoma (GBM; incidence 3.2 cases per 100,000 individuals per year) is the most malignant and aggressive one. Due to its molecular heterogeneity, high permeability and rich angiogenesis, it remains resistant to conventional treatment options, including combined surgical resection with radiotherapy and chemotherapy (with efficacy further limited by weak penetration through BBB). New therapeutic strategies are extensively being searched, aimed at preventing recurrence of tumor, prolonging survival time and improving life quality of the patients. (i.a. with the use of additional compounds improving penetration and effectiveness of chemotherapeutics) [191,192].

Tea catechins, especially EGCG, were shown to exhibit pro-apoptotic, antioxidative, anti-proliferative and anti-angiogenic properties, targeted at multiple aspects of neoplasm expansion (evasion of growth suppressors, resistance to cell death, replicative immortality and reprogramming of energy metabolism). Impact of catechins was demonstrated on a range of signaling pathways contributing to proliferation of neoplastic tumors (i.a. Janus tyrosine kinase/signal transducer and activator of transcription—JAK/STAT, Epidermal Growth Factor Receptor—tyrosine-kinase inhibitors—EGFR-TKI, Mitogen-Activated Protein Kinase—MAPK). Other relevant mechanisms of catechins anti-cancer potential include epigenetic modifications and the induction of shortening of telomeres in neoplastic cells, as well as inhibition of angiogenesis and metastasis. These effects were demonstrated on several types of cancer, with scarce but relevant evidence of EGCG potential role in brain tumors [23,36].

In the study by Grube et al., effect of EGCG extract was investigated on human glioblastoma cell cultures. While smaller concentration of EGCG did not affect glioblastoma cell proliferation (supposedly stimulating endogenous cellular repair mechanisms), the exposure to higher amounts resulted in prompt accumulation of autophagic vacuoles and increased production of ROS (cytotoxic effect) [193]. In another experimental study, liposomal preparation of polyphenols (EGCG, curcumin and resveratrol) was applied to cultured GL261 mouse GBM cells. Following this, increased apoptosis of GBM and GBM stem cells was observed (through activation of p53 tumor suppressor protein). Moreover, transformation of microglial/macrophages phenotypes was revealed (from tumor-associated M2 phenotype to contradictory M1), as well as intra-tumor activation of natural killer (NK) cells [194].

Impact upon invasiveness and migration of neoplastic cells in vitro has also been revealed for KPF. On exposure of GBM GL-15 cell line to KPF, diminished expression and activity of metalloproteinases (MMP2 and MMP9) were shown, as well as an intra- and extracellular increase in fibronectin expression and intracellular expression of laminin. Observed morphological changes in GBM cells included damage to mitochondria and rough endoplasmic reticulum and a reduction in filopodia-like structures, contributing to disturbed energetic metabolism and apoptotic processes, ultimately leading to impaired migration and viability of cells [195]. Lin et al. reported that KPF applied to GBM8401 cell line, previously exposed to 12-otetradecanoylpholia-13-acetate (a MMP9 inducer), significantly reduced MMP expression, by inhibition of kinase Cα protein, extracellular signal-regulated kinases (ERK), and NF-κB pathways. Consequently, glioblastoma cell migration and invasion were suppressed [196].

Antioxidant and anti-inflammatory properties of phenolic acids (GA, EA) were also investigated with regard to their anti-cancer effects, including brain glioma. Pedra et al. explored GA effects on the rat GBM C6 cell line and astrocyte culture, followed by evaluation of preclinical GBM model in Wistar rats. In vitro results revealed cytotoxic impact of GA on oxidative stress and nucleotide metabolism in GMB cells, but not in astrocytes. Treatment with GA in vivo for several days caused a marked reduction in tumor volume, accompanied by decreased markers of oxidative stress and nucleotides degradation in brain tissue, but also in serum, lymphocytes and platelets [197]. In human GBM cell line, Wang et al. demonstrated that EA inhibited proliferation, viability and invasion of tumor cells by promoting pro-apoptotic protein expression (caspase-3, Bax, p53) and downregulation of anti-apoptotic proteins (survivin and Bcl-2) in a dose-dependent manner. Furthermore, EA showed anti-angiogenic effect by inactivation of MMP [198]. In the interesting series of experimental studies, Cetin et al. evaluated synergistic effects of EA and cytostatic or immunomodulating agents on rat C6 glioma cells. EA combined with irinotecan (inhibitor of the topoisomerase I enzyme) reduced glioma cells proliferation by selective elevation of E-cadherin expression and decrease in N-cadherin expression in a time-independent manner. In addition, decreased expression of VEGF was observed, suggesting an anti-angiogenic effect [199]. Combination of EA and temozolomide (most commonly used in treatment of malignant glial tumors) reduced the viability of glioma cells by downregulating expression of Multi drug resistance 1 (MDR1) and O6-methylguanine-DNA methyltransferase (MGMT) genes. Enhancement of the p53 and caspase-3 proteins immunoreactivities was also observed, but without changes in gene expression. Moreover, EA alone significantly downregulated expression of MGMT and up-regulated thatof p53 and caspase-3 at selected times of exposure [200]. Similar results were demonstrated for EA combined with bevacizumab (humanized monoclonal antibody against VEGF-A), which conspicuously reduced the glioma cells viability for all incubation times. The anti-tumor effects included downregulated expression of MGMT and MDR1 and time-dependent enhancement of apoptotic activity of p53 and caspase-3 (at protein, but not at gene level), with confirmed role of EA alone in the latter [201].

Despite consistent and promising findings of anti-tumor effects of the tea compounds in the above in vitro and preclinical studies, further investigation is warranted to determine their therapeutic and preventive potential with regard to clinical management of brain tumors.

## 5. Discussion

### 5.1. Main Trends and Limitations of Investigations

Review of this extensive research on impact of tea-derived compounds upon CNS functions (in physiological conditions and in particular disorders) provides a variety of findings and evokes some interesting observations.

Firstly, there is a substantial disproportion in research evidence available for different CNS diseases. While numerous studies (experimental and clinical) were dedicated to potential role of tea and its compounds in prevention of neurodegenerative disorders (dementia, Parkinson disease), much smaller amount concerned stroke (except for general prevention of cardiovascular diseases) and even less—brain tumors. Possible reasons might be associated with lack of effective therapies for some disorders, prompting intensive search for new options, or—on the other hand—with barriers to appropriate design and conduction of clinical trials (for logistic or ethical reasons). Moreover, some of the interventional studies were terminated due to failure to reach expected endpoints, or their results have not been published after completion of the trial (Table 3, Table 4 and Table 5).

Secondly, epidemiological studies on the role of regular tea intake in prevention of risk of incidence and/or severity of particular neurological conditions brought ambiguous results [89,91,94]. It seems that more conclusive findings were impeded with by marked inter-study variability and some methodological shortcomings (e.g., cross-sectional design, short follow-up period, small sample size), which is worth considering in the interpretation of results. The history of tea intake collected retrospectively might have been burdened with selection/recall bias; regular reports/diaries would be more reliable but should contain precise quantification of tea consumption (volume, frequency) due to dose-dependent effects of its bioactive compounds. No direct comparisons have been made between the effects of particular types of tea, while their popularity and modes of beverage preparation show great diversity across the world, due to multiple cultural, regional or individual determinants [6,35]. Furthermore, there are numerous confounding factors affecting bioavailability of tea and its compounds. Apart from demographic data (age, sex, race/ethnicity), smoking, alcohol intake, dietary habits and other lifestyle factors should also be included in the multidimensional analyses. Finally, the unification of outcome measures (e.g., epidemiological indices, neuropsychological tests results, or functional scales specific for particular disorders) would allow more consistent and comparable findings from different studies [6,36]. All these aspects are worth considering in comprehensive design of future observational studies in this field.

Another striking observation is associated with the fact that only a small proportion of beneficial effects of tea compounds, demonstrated in experimental models, has been confirmed in human studies. Results from in vitro investigations can be hardly translated into in vivo/ex vivo ones for a variety of reasons [202,203]. They include extensive gut processing and metabolism of tea compounds after oral intake, leading to their rapid degradation and loss of efficacy; with exposure of tissues to metabolites rather than primary substances. Instability of these molecules in various physiological environments, especially with regard to penetration through BBB, results in their relatively low bioavailability. With dose-dependent effects of tea polyphenols revealed in many experimental models, it has to be considered that concentrations of these substances were usually much higher than those contained in tea beverages. Due to lack of standardized formulations of tested compounds (e.g., catechins), their types, doses and purity widely varied across the studies, which further impeded replication of the findings [23]. In addition, synergistic interactions among multiple tea compounds might play a relevant role in their final activity (greater than for isolated substances), in terms of beneficial but also adverse effects. Finally, animal models of CNS disorders only partially correspond with human conditions, due to obvious disparities in genetics, immune responses, metabolism, architecture and functionality of neural structures across species [6,39,204].

Thus, future scopes of research should focus on overcoming these barriers towards greater relevance of findings.

### 5.2. Future Directions of Investigations

One of promising directions of research is aimed at increasing the content of most beneficial compounds in tea beverage, with proper balance between the efficiency of the dose and its toxicity [205]. Desired content of tea leaves could be achieved through impact on the expression and activity of the key enzymes responsible for the synthesis and metabolism of L-theanine and caffeine (N-methyltransferase, N-methylnucleoside hydrolase and caffeine synthetase) [39]. These can be affected by appropriate growing methods (light exposure during growth, ambient temperature or the age-related tenderness of the leaves at harvest) [206]. Another approach could be associated with genetic engineering, including activation of the ubiquitin system related to protein degradation, destruction of chloroplast structure, inhibition of photosynthesis, and disruption of carbon/nitrogen balance [207]. Attempts to improve tea enrichment in particular substances can also be made at the stage of tea manufacturing. Instead of traditional extraction (leading to loss of some active ingredients), ultrafine grinding technology could be implemented, which allows reduction in tea particle size with enhanced solubility and absorption of beneficial tea compounds [208].

Another direction of future investigations aims at providing better and more targeted access of beneficial tea compounds to CNS, with the use of nanotechnology strategies. Encapsulating bioactive molecules in nanoparticles enhances their stability in plasma after oral administration and their overall bioavailability. The efficiency of targeted nanoparticles delivery and controlled release of their content depends on their physical and chemical properties [209]. Experimental studies showed that oral administration of EGCG encapsulated in chitosan-tripolyphosphate nanoparticles resulted in increased catechin concentrations in the animals’ gastrointestinal tract and plasma, as well as improved paracellular transport due to the transient opening of tight junctions [19,210]. Modification of nanoparticles’ surface (using different molecules, e.g., folic acid, cholesterol, phosphatidylcholine, polyethylene glycol, peptides or oligonucleotides) may prolong their circulation time in the blood, allow binding to the cell receptors (to ensure targeted delivery) and elevate cellular uptake of the encapsulated compound [211,212]. Adequate choice of nanoparticles for their content may further improve their efficacy. Hydrophilic tea compounds (e.g., catechins) could be optimally transported inside the hydrophilic core of the liposomes, while hydrophobic ones (quercetin) could be incorporated into phospholipid tails of the lipid bilayer or other nanostructured lipid carriers [213,214,215]. Tea compounds could also be encapsulated in micelles, nano-emulsions or double emulsions, or polymeric nanoparticles, such as poly(lactide-coglycolide) [212,213]. Interesting new opportunities are associated with concept of co-delivery of active substances within nanoparticles (e.g., complex of various tea polyphenols or their combination with anti-cancer or vasoactive medications) [212].

Modern biotechnological methods could be applied to solve aforementioned problems with translation of experimental findings (e.g., neuroprotective effects of tea compounds) into human physiology. Recently, organoids, the three-dimensional structures derived from human pluripotent stem cells, have been emerging as a novel tool for reproducing main aspects of human brain composition, organization and functionality [204]. Neurons within organoids exhibit signs of migration, polarity and electrical activity; moreover, development of glial cells can also be featured [216,217,218]. Thus, organoids allow the recreation of human brain environment which resembles in vivo structures more than traditional neuronal cell cultures, with regard to both to physiological conditions and models of particular CNS disorders [204,216]. Due to their characteristics, human brain organoids seem to be a useful model for testing effects of bioactive substances on CNS function, including tea compounds.

The rapid development of artificial intelligence (AI) is opening new perspectives for drug discovery and investigations of therapeutic potential of natural bioactive compounds. Recent advances in molecular modeling and computational drug screening allow researchers to predict desired activities of tested substances, elucidate their interactions with molecular targets and identify the most promising objects for further investigation [219,220]. Such methods as computational molecular docking analysis and molecular dynamics simulations can be particularly helpful in that process [35]. Molecular docking is based on computational simulation and used to predict the binding modes and affinities between small molecules and their receptors/targets [221]. Molecular dynamics simulations can further verify and optimize these binding modes, providing a better understanding of their interactions on a molecular level [222]. With the use of these methods, interesting phenomena have been revealed i.a. for the interactions between EGCG and Aβ. Pathological aggregation of Aβ (due to the increase in β-strand conformation content and exclusion of water from its surface) was efficiently prevented by an excess concentration of EGCG, while direct binding of EGCG to amino acid residues of Aβ played a minor role [223]. AI supported machine learning algorithms with virtual screening could be used to integrate data from physicochemical analysis of tested compounds with experimental findings from their biological effects, to predict the most relevant interactions with targets involved, e.g., in oxidative stress or neuroinflammation. Furthermore, AI may support the assessment of pharmacokinetic properties of tested compounds. Such analysis according to ADMET (absorption, distribution, metabolism, excretion, toxicity) scheme, could optimize evaluation of their bioavailability, metabolic stability, potential toxicity and the likelihood of BBB penetration [219,220].

Further implementation of these up-to-date methods in the studies on bioactive tea compounds might provide innovative insight into their role in preventing or counteracting processes of CNS damage, with valuable implication for therapeutic strategies.

## 6. Conclusions

Regular intake of tea, especially its types abundant in polyphenols, seems beneficial for general health, in particular preventing cardiovascular system diseases and their neurological complications, and reducing the risk of some neurodegenerative disorders. There is a range of evidence for neuroprotective and anti-inflammatory effects of tea compounds, demonstrated on experimental models of the CNS disorders. However, clinical trials and observational studies in humans often failed to confirm these effects and their results were limited to amelioration of selected aspects of diseases. Further comprehensive investigations are warranted, involving modern technologies, to prove the role of tea compounds as new therapeutic opportunities targeting background of CNS disorders.

## Figures and Tables

**Table 1:** Bioactive compounds in different types of tea. (− lack of compound, + low amount, ++ moderate amount, +++ high amount of compound).

Types of Tea		Green	White	Yellow	Oolong	Black	Pu-erh
**Processing regimens**		Panning/steaming → rolling → drying. No fermentation	Drying. No fermentation.	Steaming/panning → sweltering → rolling → drying. No fermentation.	Panning → rolling → drying → frying. Short fermentation.	Rolling → drying. Full fermentation.	Withering → pan-frying → rolling → sun drying → microbial post-fermentation → fermentation → final drying Post fermentation.
	**Content**						
**Catechins**		+++	+	++	++	+	+
**Flavonol glycosides**		+	++	++	++	+	+
**Phenolic acids**		+	+	+	++	++	+++
**Theaflavins**		−	−	−	−	+++	+
**L-theanine**		++	++	+++	++	++	+
**Caffeine**		+++	++	+++	++	++	+++

**Table 2:** Effects of major tea compounds upon CNS neurotransmitters.

	Molecular Target	Functional Effect	Clinical Implications
**EGCG**	↓ AChE, ↑ ChAT activity ↓ DA transporter ↓ MAO-B and COMT activity Small doses: ↑ GABA agonists effect, high doses: ↓ GABA agonists effect (second-order modulator)	Increased cholinergic transmission Increased dopaminergic transmission. Modulation of GABA inhibitory effects.	Improvement of cognitive functions. Regulation of movement control. Reduction in stress, calming effect.
**Caffeine**	Antagonist of adenosine receptors ↓ number of Ach receptors Modified sensitivity of cholinergic neurons ↑ ACh turnover ↑ synthesis and turnover of NA ↑ synthesis and local release of DA ↑ release of 5 HT ↓ availability of 5HT postsynaptic receptors ↓ release of GABA and glycine ↑ glutamine activity	Suppressed cholinergic transmission. Enhanced activity of noradrenergic neurons. Enhanced dopaminergic transmission. Increased cerebral concentration of serotonin. Increased excitability of neural structures	Mild stimulation of psychomotor activity (increased alertness, shortened reaction time, reduced fatigue, boosted motor and mental performance)
**L-Theanine**	Competitive agonist of NMDA-R ↓ release of glutamate ↑ release of glycine, GABA, DA, 5-HT	Enhanced inhibitory effect on neural structures. Modulated dopaminergic and serotoninergic transmission.	Reduction in stress and anxiety, promoting sleep quality, supporting cognitive performance.

Abbreviations: 5-HT—serotonin, Ach—acetylcholine, AChE—acetylcholinesterase, ChAT—choline acetylase transporter, COMT—catechol-O-methyltransferase, DA—dopamine, EGCG—epigallocatechin gallate, GABA—gamma-aminobutyric acid, MAO-B—monoamine oxidase B, NA—noradrenalin, NMDA—R—N-methyl-D-aspartate receptor). ↓—decrease; ↑—increase.

**Table 3:** Clinical trials investigating effects of tea compounds on the dementive disorders (based on Clinicaltrials.gov).

Trial Number	Title and Type of Study	Aim of the Study	Interventions	Results	Status	Reference
NCT06507254	MAEVE: Microbiota Mediated Flavonoid Metabolites for Cognitive Health. Phase 2 randomized, double-blind placebo-controlled multicenter trial.	Establishing the protective effects of regular dietary polyphenol intake on cognitive function and on brain–gut microbiome interactions, to prevent delaying the onset of AD in at-risk elderly.	Juice Plus+ (polyphenol supplement) vs. placebo; Subjects: n = 300 + validation sample: n = 100, age: ≥50 yo; Duration: 12 months; Outcome measures: analysis of blood and stool (polyphenol-derived metabolite concentrations and microbiome levels), battery of cognitive tests, online questionnaires (about diet, mood and behavior), physical body measures, brain MRI scans	Ongoing.	Recruiting	
NCT03286608	Pattern of Polyphenol Intake and the Long-term Risk of Dementia in Older Persons Observational study.	Investigation of the association between polyphenols intake and the risk of developing dementia	Observational study (no intervention); Subjects: n = 1329 age: ≥65 yo, initially without dementia; Duration: 12 years; Outcome measures: nutrient and polyphenol intake assessed based on a 24 h dietary recall, collection of sociodemographic lifestyle information, genotyping for APOE-ε4 allele, a battery of cognitive tests	Polyphenol pattern provided by a diet containing specific plant products accompanied by red wine and tea was associated with lower dementia risk.	Completed	[98]
NCT03978052	Prevention of Cognitive Decline in ApoE4 Carriers With Subjective Cognitive Decline After EGCG and a Multimodal Intervention. Two-arm randomized, double-blind, placebo-controlled clinical study.	Investigating whether a multimodal lifestyle change intervention combined with EGCG intake will slow down cognitive impairment and improve brain connectivity in a population of participants with subjective cognitive decline.	EGCG (400–600 mg/day) vs. placebo + multimodal lifestyle personalized intervention; Subjects: n = 150, age: 60–80 yo, with subjective cognitive decline, carrying the APOE-ε4 allele; Duration: 12 months + 3-month follow up; Outcome measures: battery of cognitive tests, quality of sleep (PSQI, ESS), functional and structural brain MRI; blood, urine, feces and oral fluid analysis (dietary compounds, microbiota changes; AD biomarkers: NfL, GFAP, tau protein, β-amyloid)	Cognitive improvements in the group treated with EGCG compared to placebo at 12 months approached statistical significance; by 15 months some significant cognitive benefits were noted in this group.	Completed	[99]
NCT01594086	Effects of Green Tea Consumption on Homocysteine Metabolism and Cognitive Dysfunction in Elderly: An Exploratory Clinical Study. Open-label pilot study	To investigate the effects of green tea consumption on homocysteine metabolism and cognitive dysfunction in elderly with impaired cognitive function	Green tea powder (catechins: 227 mg/day; theanine 42 mg/day); Subjects: n = 12, age: ≥65; Duration: 3 months; Outcome measures: MMSE, blood and serum analysis	Three months of daily consumption of catechins and theanine in green tea improved cognitive dysfunction, especially short-term memory.	Completed	[100]
NCT00951834	Sunphenon EGCG (Epigallocatechin-Gallate) in the Early Stage of Alzheimer’s Disease. Exploratory pilot study.	To evaluate EGCG influence (as add-on therapy) on progression of Alzheimer’s Disease	EGCG (200–800 mg/day) vs. placebo add-on to donepezil; Subjects: n = 21, age: ≥60 yo; Duration: 18 months; Outcome measures: time to hospitalization, cognitive tests, brain atrophy indices in MRI	Not provided.	Completed	
NCT04570085	Evaluating the Effect of a 30-week Caffeine Treatment on Cognition in Alzheimer’s Disease at Beginning to Moderate Stages. Phase 3 randomized, double-blind, placebo-controlled multicenter trial.	To evaluate caffeine efficacy on cognitive decline in mild to moderate stage of Alzheimer’s disease	Caffeine (400 mg/day) vs. placebo; Subjects: n = 248, age: ≥50 yo, with mild to moderate dementia in Alzheimer’s disease (MMSE 16–24); Duration: 36 weeks; Outcome measures: a battery of cognitive tests (QoL AD), sleep quality (ESS), β-amyloid and tau protein levels in CSF	Ongoing.	Recruiting	

Abbreviations: AD—Alzheimer’s Disease; CSF—Cerebrospinal fluid; ESS—Epworth Sleepiness Scale; GFAP—glial fibrillary acidic protein; MMSE—Mini Mental State Examination; MRI—Magnetic Resonance Imaging; PSQI—Pittsburgh Sleep Quality Inventory; QoL AD—quality of life in AD.

**Table 4:** Clinical trials investigating effects of tea compounds in the movement disorders: Parkinson disease and Huntington disease (based on Clinicaltrials.gov).

Trial Number	Title and Type of Study	Aim of the Study	Interventions	Results	Status	Reference
NCT01738178	Caffeine as a Therapeutic Agent in Parkinson’s Disease. Multicenter, placebo-controlled study.	To evaluate the efficacy of caffeine in relieving motor and non-motor aspects of Parkinson’s disease	Caffeine (400 mg/day) vs. placebo; Subjects: n = 118, age: 45–75 yo, with PD Hoehn and Yahr stage I-III; Duration: up to 18 months; Outcome measures: clinical assessment—MDS-UPDRS-III, MoCA, sleep quality (SCOPA-sleep, RBD1Q), BDI, and quality of life (EQ-5D)	Caffeine did not provide clinically important improvement in motor manifestations of PD. A slight improvement in somnolence over the first 6 months was noted, which attenuated over time. There was a slight increase in occurrence of dyskinesia and decline in performance of cognitive tests.	Completed	[130]
NCT01190735	Caffeine for Motor Manifestations of Parkinson’s Disease. Phase 2 open-label dose–response study.	To identify the optimal caffeine dose with maximal motor benefit and the least amount of undesirable adverse effects in PD patients	Synthetic caffeine (weekly increasing dose: 100 to 500 mg/day); Subjects: n = 28, age: ≥18 yo, with Parkinson’s disease (stage I–IV Hoehn and Yahr); Duration: 6 weeks; Outcome measures: clinical assessment—UPDRS, TUG, sleep quality (ESS, PSQI), fatigue (FSS), quality of life (PDQ-39), mood (BDI)	Not provided.	Completed	
NCT00459420	Caffeine for Excessive Daytime Somnolence in Parkinson’s Disease. Randomized, placebo-controlled study.	To assess the effect of caffeine on sleepiness and motor symptoms in patients with Parkinson’s disease	Caffeine (400 mg/day) vs. placebo; Subjects: n = 58, age: ≥18 yo, with PD; Duration: 3 weeks; Outcome measures: clinical assessment (UPDRS), sleep quality (PSQI), fatigue (FSS), mood (BDI), quality of life (PDQ-39, SF-36)	Caffeine provided only equivocal borderline improvement in excessive somnolence in PD, but improved objective motor measures.	Completed	[129]
NCT01357681	Effects of EGCG (Epigallocatechin Gallate) in Huntington’s Disease The ETON-Study. Randomized, double-blind, stratified, placebo-controlled multicenter trial.	Evaluating the changes in cognitive function in Huntington’s Disease after EGCG administration	EGCG (1200 mg/day) vs. placebo; Subjects: n = 54, age: ≥18 yo, with Huntington disease; Duration: 12 months; Outcome measures: motor function—UHDRS, Qmotor, mental health (MMSE, BDI, CGI, PANAS, Tonic and phasic Alertness), quality of life (SF-36, SWLS), brain atrophy indices in MRI, determination of huntingtin expression levels	Not provided.	Completed	
NCT03034122	Is Caffeine an Environmental Modifier in Huntington’s Disease? Observational study.	Evaluation of caffeine consumption on progression in HD	Observational study (no intervention); Subjects: n = 77, age: ≥21 yo, with HD; Duration: 2 years; Outcome measures: clinical assessment (UHDRS), atrophy indices in MRI, battery of cognitive tests, sleep quality (ESS).	Not provided.	Completed	

Abbreviations: BDI—Beck Depression Inventory; EQ-5D—EuroQoL 5-dimension; ESS—Epworth Sleepiness Scale; FSS—Fatigue Severity Scale; HD—Huntington’s Disease; MDS-UPDRS-III—Movement Disorder Society-sponsored Unified Parkinson’s Disease Rating Scale; MMSE—Mini Mental State Examination; MoCA—Montreal Cognitive Assessment; MRI—Magnetic Resonance Imaging; PANAS—Positive and Negative Affect Schedule; PD—Parkinson’s Disease; PDQ—The Parkinson’s Disease Questionnaire; PSQI—Pittsburgh Sleep Quality Inventory; Qmotor—Quantitative evaluation of motor functions; RBD1Q—REM Sleep Behavior Disorder Single-Question Screen; SCOPA—Scales for Outcomes in PD; SF-36—Short Form (36) Health Survey; SWLS—Satisfaction With Life Scale; TUG—Timed Up and Go; UHDRS—Unified Huntington’s Disease Rating Scale; UPDRS—Unified Parkinson Disease Rating Scale.

**Table 5:** Clinical trials investigating effects of tea compounds on multiple sclerosis (based on Clinicaltrials.gov).

Trial Number	Title and Type of Study	Aim of the Study	Interventions	Results	Status	Reference
NCT00836719	Safety and Neuroprotective Effects of Polyphenon E in Multiple Sclerosis. (Phase I: single group futility study)	Assessment of safety of Polyphenon E in MS and its futility as neuroprotective agent (Phase I)	Polyphenon E—green tea extract (EGCG: 800 mg/day) vs. placebo; Subjects: n = 11, age: 18–60 yo, relapsing-remitting or secondary progressive MS; Duration: 6 months (Phase I); Outcome measures: plasma levels of EGCG, N-acetyl aspartate (NAA)/creatine (Cr) levels in MRS	Polyphenon E is not futile at increasing brain NAA. Some doses of Polyphenon E have a high risk of hepatotoxicity.	Completed	[146]
NCT01451723	Safety and Neuroprotective Effects of Polyphenon E in Multiple Sclerosis. (Phase II: randomized placebo-controlled trial)	To assess neuroprotective effects of polyphenon E in patients with MS (Phase II)	Polyphenon E—green tea extract (EGCG: 800 mg/day) vs. placebo (sugar); Subjects: n = 12, age: 18–60 yo; relapsing-remitting or secondary progressive MS; Duration: 1 year (Phase II); Outcome measures: plasma levels of EGCG, N-acetyl aspartate (NAA)/creatine (Cr) levels in MRS, brain atrophy in MRI	The study was terminated due to unusual high frequency of elevated liver function tests.	Terminated	[146]
NCT01417312	Metabolic Effects of a Green Tea Extract in Multiple Sclerosis Patients. Randomized, double-blind, placebo-controlled, crossover trial.	To investigate EGCG effect on improvement of systemic and tissue energy metabolism and metabolic capacity at rest and during exercise in patients with MS.	94% EGCG green tea extract (600 mg/day) vs. placebo; Subjects: n = 18, age: 20–60 yo subjects with MS; Duration: 12 weeks; Outcome measures: fasting and postprandial increase in fat oxidation and improved efficiency of exercise muscle work assessed by indirect calorimetry	EGCG given to patients with MS over 12 weeks improves muscle metabolism during moderate exercise, to a greater extent in men than in women.	Completed	[147]
NCT00525668	Sunphenon EGCG (Epigallocatechin-gallate) in Relapsing-remitting Multiple Sclerosis (SuniMS Study) Phase 2 randomized, double-blind, placebo-controlled trial.	To assess anti-inflammatory and neuroprotective properties of green tea extract with 95% EGCG in patients with relapsing-remitting multiple sclerosis (RRMS)	Sunphenon—95% EGCG green tea extract (800 mg/day) vs. placebo as an add-on to GlAc; Subjects: n = 70, age: 18–60 yo, with relapsing-remitting MS; Duration: 18 months; Outcome measures: clinical assessment—EDSS, MSFC, brain lesions and atrophy in MRI	Oral EGCG add-on to GA did not influence MRI and clinical disease activity and immune parameters over 18 months. The treatment was safe at a daily dosage up to 800 mg EGCG.	Completed	[148]
NCT00799890	Evaluating the Effect of Sunphenon EGCG (Main Component Epigallocatechin-Gallate) on the Increase in Brain Atrophy in the Cerebral Magnetic Resonance Tomography in a 36 months Treatment Time in Patients With Primary or Secondary Chronic-progressive Multiple Sclerosis. Two-arm randomized/stratified, double-blind placebo-controlled study.	To assess Sunphenon (EGCG) anti-inflammatory and neuroprotective potence in MS treatment	Sunphenon—95% extract of EGCG (200–1200 mg/day) vs. placebo; Subjects: n = 37, age: 18–65 yo, primary or secondary progressive MS; Duration: 36 months with an optional open-label extension for 12 months; Outcome measures: clinical assessment—EDSS, MSFC, fatigue (FSS, MSFC), mood (BDI), brain atrophy indices in MRI, OCT	There was no evidence of EGCG treatment effect on the radiologic and clinical measures in progressive forms of MS.	Completed	[149]
NCT03740295	Impact of Ketone Bodies and Epigallocatechin Gallate in Multiple Sclerosis. Exploratory pilot study.	To assess the impact of a nutritional intervention [isocaloric Mediterranean diet] on the evolution of MS patients in their different slopes	EGCG (800 mg/day) and coconut oil (60 mL/day) (3600 mg of TGCM) vs. placebo as an add-on to isocaloric Mediterranean diet; Subjects: n = 46, age: 18–65 yo, with MS; Duration: 4 months; Outcome measures: clinical assessment (EDSS, functional motor tests), mental health (BDI, STAI), anthropometric measurements, pro- and antioxidative parameters in blood tests	Administration of EGCG to MS patients increased the serum level of ketone bodies, PON1 and albumin and improved anthropometric measures (reduction in WHR and an increase in muscle percentage).	Completed	[150]

Abbreviations: BDI—Beck Depression Inventory; CGI—Clinical Global Impression; EDSS—Expanded Disability Status Scale; FSS—Fatigue Severity Scale; GlAc—glatiramer acetate; MRI—Magnetic Resonance Imaging; MRS—Magnetic resonance spectroscopy; MS—Multiple Sclerosis; MSFC—Multiple Sclerosis Functional Composite; OCT—Optical Coherence Tomography; PON1—Paraoxonase 1; STAI—State Trait Anxiety Inventory.

## Data Availability

No new data were created or analyzed in this study. Data sharing is not applicable to this article.

## References

[1] Castillo-Ruiz M., Espinoza J.P., Benavides L., Otero M.C. (2025). Biological Activities of Tea: Benefits, Risks, and Critical Overview of Their Consumption in Children. Beverages.

[2] Das C., Banerjee A., Saha M., Chatterjee S. (2022). A Review of the Health Benefits of Tea: Implications of the Biochemical Properties of the Bioactive Constituents. Curr. Res. Nutr. Food Sci. J..

[3] Ferrara L., Montesano D., Senatore A. (2001). The distribution of minerals and flavonoids in the tea plant (*Camellia sinensis*). Il Farm..

[4] Tang G.-Y., Meng X., Gan R.-Y., Zhao C.-N., Liu Q., Feng Y.-B., Li S., Wei X.-L., Atanasov A.G., Corke H. (2019). Health Functions and Related Molecular Mechanisms of Tea Components: An Update Review. Int. J. Mol. Sci..

[5] Khan N., Mukhtar H. (2013). Tea and Health: Studies in Humans. Curr. Pharm. Des..

[6] Piyasena K.G.N.P., Napagoda M.T., Kalinga J., Jayasinghe L. (2026). Health benefits of different types of tea: A review. Food Nutr. Health.

[7] Luo Q., Luo L., Zhao J., Wang Y., Luo H. (2024). Biological potential and mechanisms of Tea’s bioactive compounds: An Updated review. J. Adv. Res..

[8] Feigin V.L., Vos T., Nichols E., O Owolabi M., Carroll W.M., Dichgans M., Deuschl G., Parmar P., Brainin M., Murray C. (2020). The global burden of neurological disorders: Translating evidence into policy. Lancet Neurol..

[9] Fernandes L., Cardim-Pires T.R., Foguel D., Palhano F.L. (2021). Green Tea Polyphenol Epigallocatechin-Gallate in Amyloid Aggregation and Neurodegenerative Diseases. Front Neurosci..

[10] Gonçalves P.B., Sodero A.C.R., Cordeiro Y. (2021). Green Tea Epigallocatechin-3-gallate (EGCG) Targeting Protein Misfolding in Drug Discovery for Neurodegenerative Diseases. Biomolecules.

[11] Wang S., Jiang Y., Yang A., Meng F., Zhang J. (2024). The Expanding Burden of Neurodegenerative Diseases: An Unmet Medical and Social Need. Aging Dis..

[12] Kochman J., Jakubczyk K., Antoniewicz J., Mruk H., Janda K. (2020). Health Benefits and Chemical Composition of Matcha Green Tea: A Review. Molecules.

[13] Yang M., Zhang X., Yang C.S. (2025). Bioavailability of Tea Polyphenols: A Key Factor in Understanding Their Mechanisms of Action In Vivo and Health Effects. J. Agric. Food Chem..

[14] Anandh Babu P., Liu D. (2008). Green Tea Catechins and Cardiovascular Health: An Update. Curr. Med. Chem..

[15] Singh B.N., Shankar S., Srivastava R.K. (2011). Green tea catechin, epigallocatechin-3-gallate (EGCG): Mechanisms, perspectives and clinical applications. Biochem Pharmacol..

[16] Wong M., Sirisena S., Ng K. (2022). Phytochemical profile of differently processed tea: A review. J. Food Sci..

[17] Saklar S., Ertas E., Ozdemir I.S., Karadeniz B. (2015). Effects of different brewing conditions on catechin content and sensory acceptance in Turkish green tea infusions. J. Food Sci. Technol..

[18] Jin Y., Zhao J., Kim E.M., Kim K.H., Kang S., Lee H., Lee J. (2019). Comprehensive Investigation of the Effects of Brewing Conditions in Sample Preparation of Green Tea Infusions. Molecules.

[19] Dube A., Nicolazzo J.A., Larson I. (2011). Chitosan nanoparticles enhance the plasma exposure of (−)-epigallocatechin gallate in mice through an enhancement in intestinal stability. Eur. J. Pharm. Sci..

[20] Mereles D., Hunstein W. (2011). Epigallocatechin-3-gallate (EGCG) for Clinical Trials: More Pitfalls than Promises?. Int. J. Mol. Sci..

[21] Weinreb O., Amit T., Mandel S., Youdim M.B.H. (2009). Neuroprotective molecular mechanisms of (−)-epigallocatechin-3-gallate: A reflective outcome of its antioxidant, iron chelating and neuritogenic properties. Genes Nutr..

[22] Truong V.-L., Jeong W.-S. (2021). Cellular Defensive Mechanisms of Tea Polyphenols: Structure-Activity Relationship. Int. J. Mol. Sci..

[23] Fokou P.V.T., Pone B.K., Appiah-Oppong R., Ngouana V., Bakarnga-Via I., Woutouoba D.N., Donkeng V.F.D., Yamthe L.R.T., Boyom F.F., Ateşşahin D.A. (2025). An Update on Antitumor Efficacy of Catechins: From Molecular Mechanisms to Clinical Applications. Food Sci. Nutr..

[24] Lin L., Zeng L., Liu A., Peng Y., Yuan D., Zhang S., Li Y., Chen J., Xiao W., Gong Z. (2020). l-Theanine regulates glucose, lipid, and protein metabolism *via* insulin and AMP-activated protein kinase signaling pathways. Food Funct..

[25] Farias-Pereira R., Park C.-S., Park Y. (2019). Mechanisms of action of coffee bioactive components on lipid metabolism. Food Sci. Biotechnol..

[26] Misaka S., Kawabe K., Onoue S., Werba J.P., Giroli M., Tamaki S., Kan T., Kimura J., Watanabe H., Yamada S. (2013). Effects of Green Tea Catechins on Cytochrome P450 2B6, 2C8, 2C19, 2D6 and 3A Activities in Human Liver and Intestinal Microsomes. Drug Metab. Pharmacokinet..

[27] Satoh T., Fujisawa H., Nakamura A., Takahashi N., Watanabe K. (2016). Inhibitory Effects of Eight Green Tea Catechins on Cytochrome P450 1A2, 2C9, 2D6, and 3A4 Activities. J. Pharm. Pharm. Sci..

[28] Hu J., Webster D., Cao J., Shao A. (2018). The safety of green tea and green tea extract consumption in adults—Results of a systematic review. Regul. Toxicol. Pharmacol..

[29] Teschke R., Zhang L., Melzer L., Schulze J., Eickhoff A. (2014). Green tea extract and the risk of drug-induced liver injury. Expert Opin. Drug Metab. Toxicol..

[30] Chan P.C., Ramot Y., Malarkey D.E., Blackshear P., Kissling G.E., Travlos G., Nyska A. (2010). Fourteen-Week Toxicity Study of Green Tea Extract in Rats and Mice. Toxicol. Pathol..

[31] NTP (2016). NTP Technical Report on the Toxicology Studies of Green Tea Extract in F344/NTac Rats and B6C3F1/N Mice and Toxicology and Carcinogenesis Studies of Green Tea Extract in Wistar Han [Crl:Wi(Han)] Rats and B6C3F1/N Mice (Gavage Studies). 111 TW Alexander Dr, Durham, NC 27709: 2016.

[32] Rauf M.Q., Sharma L., Essiet E., Elhassan O., Fahim R., Irem-Oko F., Sreedharan J. (2025). Caffeine Consumption Patterns, Health Impacts, and Media Influence: A Narrative Review. Cureus.

[33] Schwalfenberg G., Genuis S.J., Rodushkin I. (2013). The Benefits and Risks of Consuming Brewed Tea: Beware of Toxic Element Contamination. J. Toxicol..

[34] Afzal O., Dalhat M.H., Altamimi A.S.A., Rasool R., Alzarea S.I., Almalki W.H., Murtaza B.N., Iftikhar S., Nadeem S., Nadeem M.S. (2022). Green Tea Catechins Attenuate Neurodegenerative Diseases and Cognitive Deficits. Molecules.

[35] Pervin M., Unno K., Ohishi T., Tanabe H., Miyoshi N., Nakamura Y. (2018). Beneficial Effects of Green Tea Catechins on Neurodegenerative Diseases. Molecules.

[36] Sebastiani G., Almeida-Toledano L., Serra-Delgado M., Navarro-Tapia E., Sailer S., Valverde O., Garcia-Algar O., Andreu-Fernández V. (2021). Therapeutic Effects of Catechins in Less Common Neurological and Neurodegenerative Disorders. Nutrients.

[37] Kim Y., Cho A.Y., Kim H.C., Ryu D., Jo S.A., Jung Y.-S. (2022). Effects of Natural Polyphenols on Oxidative Stress-Mediated Blood-Brain Barrier Dysfunction. Antioxidants.

[38] Pun P.B.L., Lu J., Moochhala S. (2009). Involvement of ROS in BBB dysfunction. Free Radic. Res..

[39] Yue R., Wen H., He L., Liu Y., Liang Z., Luo L. (2025). Tea and Blood–Brain Barrier Homeostasis: Potential Mechanisms and Improvement Strategies. Food Front.

[40] Pervin M., Unno K., Nakagawa A., Takahashi Y., Iguchi K., Yamamoto H., Hoshino M., Hara A., Takagaki A., Nanjo F. (2017). Blood brain barrier permeability of (−)-epigallocatechin gallate, its proliferation-enhancing activity of human neuroblastoma SH-SY5Y cells, and its preventive effect on age-related cognitive dysfunction in mice. Biochem Biophys. Rep..

[41] Pervin M., Unno K., Takagaki A., Isemura M., Nakamura Y. (2019). Function of Green Tea Catechins in the Brain: Epigallocatechin Gallate and its Metabolites. Int. J. Mol. Sci..

[42] Faria A., Pestana D., Teixeira D., Couraud P.-O., Romero I., Weksler B., de Freitas V., Mateus N., Calhau C. (2011). Insights into the putative catechin and epicatechin transport across blood-brain barrier. Food Funct..

[43] Ma Q., Xing C., Long W., Wang H.Y., Liu Q., Wang R.-F. (2019). Impact of microbiota on central nervous system and neurological diseases: The gut-brain axis. J. Neuroinflamm..

[44] Láng L., McArthur S., Lazar A.S., Pourtau L., Gaudout D., Pontifex M.G., Müller M., Vauzour D. (2024). Dietary (Poly)phenols and the Gut–Brain Axis in Ageing. Nutrients.

[45] Lippolis T., Cofano M., Caponio G.R., De Nunzio V., Notarnicola M. (2023). Bioaccessibility and Bioavailability of Diet Polyphenols and Their Modulation of Gut Microbiota. Int. J. Mol. Sci..

[46] Wu G., Gu W., Chen G., Cheng H., Li D., Xie Z. (2023). Interactions of tea polysaccharides with gut microbiota and their health-promoting effects to host: Advances and perspectives. J. Funct. Foods.

[47] Yılmaz C., Özdemir F., Gökmen V. (2020). Investigation of free amino acids, bioactive and neuroactive compounds in different types of tea and effect of black tea processing. LWT.

[48] Johnston G.A.R. (2015). Flavonoid nutraceuticals and ionotropic receptors for the inhibitory neurotransmitter GABA. Neurochem Int..

[49] Singh N.A., Mandal A.K.A., Khan Z.A. (2015). Potential neuroprotective properties of epigallocatechin-3-gallate (EGCG). Nutr. J..

[50] Ferré S. (2010). Role of the Central Ascending Neurotransmitter Systems in the Psychostimulant Effects of Caffeine. J. Alzheimer’s Dis..

[51] Nehlig A., Daval J.-L., Debry G. (1992). Caffeine and the central nervous system: Mechanisms of action, biochemical, metabolic and psychostimulant effects. Brain Res. Rev..

[52] Lardner A.L. (2014). Neurobiological effects of the green tea constituent theanine and its potential role in the treatment of psychiatric and neurodegenerative disorders. Nutr. Neurosci..

[53] Sebih F., Rousset M., Bellahouel S., Rolland M., Ferreira M.C.d.J., Guiramand J., Cohen-Solal C., Barbanel G., Cens T., Abouazza M. (2017). Characterization of l-Theanine Excitatory Actions on Hippocampal Neurons: Toward the Generation of Novel *N* -Methyl-d-aspartate Receptor Modulators Based on Its Backbone. ACS Chem. Neurosci..

[54] Mátyus R.O., Szikora Z., Bodó D., Szabó B.V., Csupor É., Csupor D., Tóth B. (2025). Promising, but Not Completely Conclusive—The Effect of l-Theanine on Cognitive Performance Based on the Systematic Review and Meta-Analysis of Randomized Placebo-Controlled Clinical Trials. J. Clin. Med..

[55] Giesbrecht T., Rycroft J.A., Rowson M.J., De Bruin E.A. (2010). The combination of L-theanine and caffeine improves cognitive performance and increases subjective alertness. Nutr. Neurosci..

[56] Stefaniak O., Dobrzyńska M., Drzymała-Czyż S., Przysławski J. (2022). Diet in the Prevention of Alzheimer’s Disease: Current Knowledge and Future Research Requirements. Nutrients.

[57] Long J.M., Holtzman D.M. (2019). Alzheimer Disease: An Update on Pathobiology and Treatment Strategies. Cell.

[58] Zheng Q., Wang X. (2025). Alzheimer’s disease: Insights into pathology, molecular mechanisms, and therapy. Protein Cell.

[59] Hogan D.B., Jetté N., Fiest K.M., Roberts J.I., Pearson D., Smith E.E., Roach P., Kirk A., Pringsheim T., Maxwell C.J. (2016). The Prevalence and Incidence of Frontotemporal Dementia: A Systematic Review. Can. J. Neurol. Sci. J. Can. Des. Sci. Neurol..

[60] Hernandez I., Fernandez M.-V., Tarraga L., Boada M., Ruiz A. (2018). Frontotemporal Lobar Degeneration (FTLD): Review and Update for Clinical Neurologists. Curr. Alzheimer Res..

[61] Scholz S.W., Okubadejo N.U., Prakash P., Liddelow S.A., Ryten M., Halliday G.M. (2025). Advances in the genetics and pathology of Lewy body dementia. Lancet Neurol..

[62] Sachdev P.S., Bentvelzen A.C., Gustafson D., Hansra G.K., Hosoki S., Jiang J., Lennon M.J., Moro M.A., Saks D.G., Samaras K. (2026). Vascular Cognitive Impairment and Dementia. JACC.

[63] Alzheimer’s Association (2016). Alzheimer’s disease facts and figures. Alzheimer’s Dement..

[64] Garre-Olmo J. (2018). Epidemiology of Alzheimer’s disease and other dementias. Rev. Neurol..

[65] Jeong J.H., Kim H.J., Lee T.J., Kim M.K., Park E.S., Choi B.S. (2004). Epigallocatechin 3-gallate attenuates neuronal damage induced by 3-hydroxykynurenine. Toxicology.

[66] Rezai-Zadeh K., Shytle D., Sun N., Mori T., Hou H., Jeanniton D., Ehrhart J., Townsend K., Zeng J., Morgan D. (2005). Green Tea Epigallocatechin-3-Gallate (EGCG) Modulates Amyloid Precursor Protein Cleavage and Reduces Cerebral Amyloidosis in Alzheimer Transgenic Mice. J. Neurosci..

[67] Rezai-Zadeh K., Arendash G.W., Hou H., Fernandez F., Jensen M., Runfeldt M., Shytle R.D., Tan J. (2008). Green tea epigallocatechin-3-gallate (EGCG) reduces β-amyloid mediated cognitive impairment and modulates tau pathology in Alzheimer transgenic mice. Brain Res..

[68] Walker J.M., Klakotskaia D., Ajit D., Weisman G.A., Wood W.G., Sun G.Y., Serfozo P., Simonyi A., Schachtman T.R. (2015). Beneficial Effects of Dietary EGCG and Voluntary Exercise on Behavior in an Alzheimer’s Disease Mouse Model. J. Alzheimer’s Dis..

[69] Chen T., Yang Y., Zhu S., Lu Y., Zhu L., Wang Y., Wang X. (2020). Inhibition of Aβ aggregates in Alzheimer’s disease by epigallocatechin and epicatechin-3-gallate from green tea. Bioorg Chem..

[70] Wang I.-F., Chang H.-Y., Hou S.-C., Liou G.-G., Way T.-D., James Shen C.-K. (2012). The self-interaction of native TDP-43 C terminus inhibits its degradation and contributes to early proteinopathies. Nat. Commun..

[71] Taniguchi S., Suzuki N., Masuda M., Hisanaga S.-I., Iwatsubo T., Goedert M., Hasegawa M. (2005). Inhibition of Heparin-induced Tau Filament Formation by Phenothiazines, Polyphenols, and Porphyrins. J. Biol. Chem..

[72] Biasibetti R., Tramontina A.C., Costa A.P., Dutra M.F., Quincozes-Santos A., Nardin P., Bernardi C.L., Wartchow K.M., Lunardi P.S., Gonçalves C.-A. (2013). Green tea (−)epigallocatechin-3-gallate reverses oxidative stress and reduces acetylcholinesterase activity in a streptozotocin-induced model of dementia. Behav. Brain Res..

[73] Haque A.M., Hashimoto M., Katakura M., Hara Y., Shido O. (2008). Green tea catechins prevent cognitive deficits caused by Aβ1–40 in rats. J. Nutr. Biochem.

[74] Silva dos Santos J., Gonçalves Cirino J.P., de Oliveira Carvalho P., Ortega M.M. (2021). The Pharmacological Action of Kaempferol in Central Nervous System Diseases: A Review. Front Pharmacol..

[75] Hanaki M., Murakami K., Akagi K., Irie K. (2016). Structural insights into mechanisms for inhibiting amyloid β42 aggregation by non-catechol-type flavonoids. Bioorg Med. Chem..

[76] Yang E.-J., Kim G.-S., Jun M., Song K.-S. (2014). Kaempferol attenuates the glutamate-induced oxidative stress in mouse-derived hippocampal neuronal HT22 cells. Food Funct..

[77] Park S., Sapkota K., Kim S., Kim H., Kim S. (2011). Kaempferol acts through mitogen-activated protein kinases and protein kinase B/AKT to elicit protection in a model of neuroinflammation in BV2 microglial cells. Br. J. Pharmacol..

[78] Kouhestani S., Jafari A., Babaei P. (2018). Kaempferol attenuates cognitive deficit via regulating oxidative stress and neuroinflammation in an ovariectomized rat model of sporadic dementia. Neural Regen. Res..

[79] Beg T., Jyoti S., Naz F., Rahul X., Ali F., Ali S.K., Reyad A.M., Siddique Y.H. (2018). Protective Effect of Kaempferol on the Transgenic Drosophila Model of Alzheimer’s Disease. CNS Neurol. Disord. Drug Targets.

[80] Zarei M., Mohammadi S., Jabbari S., Shahidi S. (2019). Intracerebroventricular microinjection of kaempferol on memory retention of passive avoidance learning in rats: Involvement of cholinergic mechanism(s). Int. J. Neurosci..

[81] Islam R., Rauf A., Rahaman S., Al-Imran I.K., Fakir N.H., Akter S., Akter H., Habiba U., Hussain S., Al Abdulmonem W. (2025). Clinical and Mechanistic Exploration of Ellagic Acid in Neurodegenerative Diseases: Targeted Neuroprotection Through Cellular and Molecular Mechanisms. Chem. Biodivers..

[82] Wang W., Wang S., Liu Y., Wang X., Nie J., Meng X., Zhang Y. (2022). Ellagic Acid: A Dietary-Derived Phenolic Compound for Drug Discovery in Mild Cognitive Impairment. Front Aging Neurosci..

[83] Ogunlade B., Adelakun S.A., Agie J.A. (2022). Nutritional supplementation of gallic acid ameliorates Alzheimer-type hippocampal neurodegeneration and cognitive impairment induced by aluminum chloride exposure in adult Wistar rats. Drug Chem. Toxicol..

[84] Mansouri M.T., Farbood Y., Naghizadeh B., Shabani S., Mirshekar M.A., Sarkaki A. (2016). Beneficial effects of ellagic acid against animal models of scopolamine- and diazepam-induced cognitive impairments. Pharm. Biol..

[85] Kim H.K., Kim M., Kim S., Kim M., Chung J.H. (2004). Effects of Green Tea Polyphenol on Cognitive and Acetylcholinesterase Activities. Biosci. Biotechnol. Biochem..

[86] Kaur T., Pathak C.M., Pandhi P., Khanduja K.L. (2008). Effects of green tea extract on learning, memory, behavior and acetylcholinesterase activity in young and old male rats. Brain Cogn..

[87] Zhu G., Yang S., Xie Z., Wan X. (2018). Synaptic modification by L-theanine, a natural constituent in green tea, rescues the impairment of hippocampal long-term potentiation and memory in AD mice. Neuropharmacology.

[88] Elbaz E.M., Ibrahim S.M., Rashad E., Yasin N.A.E., Ghaiad H.R., Mehana N.A. (2025). Therapeutic Role of l -Theanine in Mitigating Cognitive Dysfunction and Neuropathology in Scopolamine-Treated Mice. ACS Chem. Neurosci..

[89] Ma Q.-P., Huang C., Cui Q.-Y., Yang D.-J., Sun K., Chen X., Li X.-H. (2016). Meta-Analysis of the Association between Tea Intake and the Risk of Cognitive Disorders. PLoS ONE.

[90] Jiang N., Ma J., Wang Q., Xu Y., Wei B. (2023). Tea intake or consumption and the risk of dementia: A meta-analysis of prospective cohort studies. PeerJ.

[91] Hu H.-Y., Wu B.-S., Ou Y.-N., Ma Y.-H., Huang Y.-Y., Cheng W., Tan L., Yu J.-T. (2022). Tea consumption and risk of incident dementia: A prospective cohort study of 377 592 UK Biobank participants. Transl. Psychiatry.

[92] Cornelis M.C., Bennett D.A., Weintraub S., Schneider J.A., Morris M.C. (2022). Caffeine Consumption and Dementia: Are Lewy Bodies the Link?. Ann. Neurol..

[93] Jin T., Lu D., Chen J., Zhao W., Zhang Y., Zhang Q., Ye X., Qin J. (2025). Moderate tea consumption and dementia-related neuroimaging markers. Front Neurol..

[94] Kim Y.-S., Kwak S.M., Myung S.-K. (2015). Caffeine Intake from Coffee or Tea and Cognitive Disorders: A Meta-Analysis of Observational Studies. Neuroepidemiology.

[95] Maia L., De Mendonça A. (2002). Does caffeine intake protect from Alzheimer’s disease?. Eur. J. Neurol..

[96] Santos C., Lunet N., Azevedo A., de Mendonça A., Ritchie K., Barros H. (2010). Caffeine Intake is Associated with a Lower Risk of Cognitive Decline: A Cohort Study from Portugal. J. Alzheimer’s Dis..

[97] Ritchie K., Carrière I., De Mendonca A., Portet F., Dartigues J.F., Rouaud O., Barberger-Gateau P., Ancelin M.L. (2007). The neuroprotective effects of caffeine. Neurology.

[98] Lefèvre-Arbogast S., Gaudout D., Bensalem J., Letenneur L., Dartigues J.-F., Hejblum B.P., Féart C., Delcourt C., Samieri C. (2018). Pattern of polyphenol intake and the long-term risk of dementia in older persons. Neurology.

[99] Forcano L., Soldevila-Domenech N., Boronat A., Sánchez-Benavides G., Puig-Pijoan A., Lorenzo T., Aldea-Perona A., Suárez-Calvet M., Cuenca-Royo A., Gispert J.D. (2025). A multimodal lifestyle intervention complemented with epigallocatechin gallate to prevent cognitive decline in APOE-ε4 carriers with Subjective Cognitive Decline: A randomized, double-blinded clinical trial (PENSA study). J. Prev. Alzheimers Dis..

[100] Ide K., Yamada H., Takuma N., Park M., Wakamiya N., Nakase J., Ukawa Y., Sagesaka Y.M. (2014). Green Tea Consumption Affects Cognitive Dysfunction in the Elderly: A Pilot Study. Nutrients.

[101] Ide K., Yamada H., Takuma N., Kawasaki Y., Harada S., Nakase J., Ukawa Y., Sagesaka Y.M. (2015). Effects of green tea consumption on cognitive dysfunction in an elderly population: A randomized placebo-controlled study. Nutr. J..

[102] Park S.-K., Jung I.-C., Lee W.K., Lee Y.S., Park H.K., Go H.J., Kim K., Lim N.K., Hong J.T., Ly S.Y. (2011). A Combination of Green Tea Extract and l -Theanine Improves Memory and Attention in Subjects with Mild Cognitive Impairment: A Double-Blind Placebo-Controlled Study. J. Med. Food.

[103] Dowding C.H., Shenton C.L., Salek S.S. (2006). A Review of the Health-Related Quality of Life and Economic Impact of Parkinson’s Disease. Drugs Aging.

[104] Tryphena K.P., Nikhil U.S., Pinjala P., Srivastava S., Singh S.B., Khatri D.K. (2023). Mitochondrial Complex I as a Pathologic and Therapeutic Target for Parkinson’s Disease. ACS Chem. Neurosci..

[105] Tang Y., Le W. (2016). Differential Roles of M1 and M2 Microglia in Neurodegenerative Diseases. Mol. Neurobiol..

[106] Xia Y., Zhang G., Kou L., Yin S., Han C., Hu J., Wan F., Sun Y., Wu J., Li Y. (2021). Reactive microglia enhance the transmission of exosomal α-synuclein via toll-like receptor 2. Brain.

[107] Postuma R.B., Berg D., Stern M., Poewe W., Olanow C.W., Oertel W., Obeso J., Marek K., Litvan I., Lang A.E. (2015). MDS clinical diagnostic criteria for Parkinson’s disease. Mov. Disord..

[108] Höglinger G., Trenkwalder C., German Parkinson’s Guidelines Committee (2024). Diagnosis and treatment of Parkinson’s disease (guideline of the German Society for Neurology). Neurol. Res. Pract..

[109] Li S., Wang Z., Liu G., Chen M. (2024). Neurodegenerative diseases and catechins: (−)-epigallocatechin-3-gallate is a modulator of chronic neuroinflammation and oxidative stress. Front Nutr..

[110] Li R., Huang Y., Fang D., Le W. (2004). (−)-Epigallocatechin gallate inhibits lipopolysaccharide-induced microglial activation and protects against inflammation-mediated dopaminergic neuronal injury. J. Neurosci. Res..

[111] Özduran G., Becer E., Vatansever H.S., Yücecan S. (2022). Neuroprotective effects of catechins in an experimental Parkinson’s disease model and SK-N-AS cells: Evaluation of cell viability, anti-inflammatory and anti-apoptotic effects. Neurol. Res..

[112] Zhou Z.D., Xie S.P., Saw W.T., Ho P.G.H., Wang H.Y., Zhou L., Zhao Y., Tan E.K. (2019). The Therapeutic Implications of Tea Polyphenols against Dopamine (DA) Neuron Degeneration in Parkinson’s Disease (PD). Cells.

[113] Siddique Y.H., Jyoti S., Naz F. (2014). Effect of Epicatechin Gallate Dietary Supplementation on Transgenic *Drosophila* Model of Parkinson’s Disease. J. Diet. Suppl..

[114] Zhou T., Zhu M., Liang Z. (2018). (-)-Epigallocatechin-3-gallate modulates peripheral immunity in the MPTP-induced mouse model of Parkinson’s disease. Mol. Med. Rep..

[115] Cheng C.-Y., Barro L., Tsai S.-T., Feng T.-W., Wu X.-Y., Chao C.-W., Yu R.-S., Chin T.-Y., Hsieh M.F. (2021). Epigallocatechin-3-Gallate-Loaded Liposomes Favor Anti-Inflammation of Microglia Cells and Promote Neuroprotection. Int. J. Mol. Sci..

[116] Shen J., Xie J., Ye L., Mao J., Sun S., Chen W., Wei S., Ruan S., Wang L., Hu H. (2024). Neuroprotective effect of green tea extract (-)-epigallocatechin-3-gallate in a preformed fibril-induced mouse model of Parkinson’s disease. Neuroreport.

[117] Tseng H.-C., Wang M.-H., Chang K.-C., Soung H.-S., Fang C.-H., Lin Y.-W., Li K.-Y., Yang C.-C., Tsai C.-C. (2020). Protective Effect of (−)Epigallocatechin-3-gallate on Rotenone-Induced Parkinsonism-like Symptoms in Rats. Neurotox. Res..

[118] Xu Q., Langley M., Kanthasamy A.G., Reddy M.B. (2017). Epigallocatechin Gallate Has a Neurorescue Effect in a Mouse Model of Parkinson Disease. J. Nutr..

[119] Yang Y.-L., Cheng X., Li W.-H., Liu M., Wang Y.-H., Du G.-H. (2019). Kaempferol Attenuates LPS-Induced Striatum Injury in Mice Involving Anti-Neuroinflammation, Maintaining BBB Integrity, and Down-Regulating the HMGB1/TLR4 Pathway. Int. J. Mol. Sci..

[120] Han X., Sun S., Sun Y., Song Q., Zhu J., Song N., Chen M., Sun T., Xia M., Ding J. (2019). Small molecule-driven NLRP3 inflammation inhibition via interplay between ubiquitination and autophagy: Implications for Parkinson disease. Autophagy.

[121] Li S., Pu X.-P. (2011). Neuroprotective Effect of Kaempferol against a 1-Methyl-4-phenyl-1,2,3,6-tetrahydropyridine-Induced Mouse Model of Parkinson’s Disease. Biol. Pharm. Bull..

[122] Gao X., Cassidy A., Schwarzschild M.A., Rimm E.B., Ascherio A. (2012). Habitual intake of dietary flavonoids and risk of Parkinson disease. Neurology.

[123] Chen H., Ding D., Wang J., Zhao Q., Meng H., Li H., Gao Y.-T., Shu X.-O., Tanner C.M., Hong Z. (2015). Parkinson’s disease research in a prospective cohort in China. Park. Relat. Disord..

[124] Qi H., Li S. (2014). Dose–response meta-analysis on coffee, tea and caffeine consumption with risk of Parkinson’s disease. Geriatr. Gerontol. Int..

[125] Hosseini Tabatabaei N. (2013). Non-genetic factors associated with the risk of Parkinson’s disease in Iranian patients. Funct. Neurol..

[126] Tanaka K., Miyake Y., Fukushima W., Sasaki S., Kiyohara C., Tsuboi Y., Yamada T., Oeda T., Miki T., Kawamura N. (2011). Intake of Japanese and Chinese teas reduces risk of Parkinson’s disease. Park. Relat. Disord..

[127] Li C., Lin J., Yang T., Shang H. (2022). Green Tea Intake and Parkinson’s Disease Progression: A Mendelian Randomization Study. Front. Nutr..

[128] Caruana M., Vassallo N. (2015). Tea Polyphenols in Parkinson’s Disease.

[129] Postuma R.B., Lang A.E., Munhoz R.P., Charland K., Pelletier A., Moscovich M., Filla L., Zanatta D., Romenets S.R., Altman R. (2012). Caffeine for treatment of Parkinson disease. Neurology.

[130] Postuma R.B., Anang J., Pelletier A., Joseph L., Moscovich M., Grimes D., Furtado S., Munhoz R.P., Appel-Cresswell S., Moro A. (2017). Caffeine as symptomatic treatment for Parkinson disease (Café-PD). Neurology.

[131] Nopoulos P.C. (2016). Huntington disease: A single-gene degenerative disorder of the striatum. Dialogues Clin. Neurosci..

[132] Ehrnhoefer D.E., Duennwald M., Markovic P., Wacker J.L., Engemann S., Roark M., Legleiter J., Marsh J.L., Thompson L.M., Lindquist S. (2006). Green tea (−)-epigallocatechin-gallate modulates early events in huntingtin misfolding and reduces toxicity in Huntington’s disease models. Hum. Mol. Genet.

[133] Beasley M., Stonebraker A.R., Hasan I., Kapp K.L., Liang B.J., Agarwal G., Groover S., Sedighi F., Legleiter J. (2019). Lipid Membranes Influence the Ability of Small Molecules To Inhibit Huntingtin Fibrillization. Biochemistry.

[134] Varga J., Dér N.P., Zsindely N., Bodai L. (2020). Green tea infusion alleviates neurodegeneration induced by mutant Huntingtin in *Drosophila*. Nutr. Neurosci..

[135] Sandhir R., Mehrotra A. (2013). Quercetin supplementation is effective in improving mitochondrial dysfunctions induced by 3-nitropropionic acid: Implications in Huntington’s disease. Biochim. Biophys. Acta (BBA)—Mol. Basis Dis..

[136] Lassmann H. (2019). Pathogenic Mechanisms Associated with Different Clinical Courses of Multiple Sclerosis. Front Immunol..

[137] Dobson R., Giovannoni G. (2019). Multiple sclerosis—A review. Eur. J. Neurol..

[138] Wang J., Ren Z., Xu Y., Xiao S., Meydani S.N., Wu D. (2012). Epigallocatechin-3-Gallate Ameliorates Experimental Autoimmune Encephalomyelitis by Altering Balance among CD4+ T-Cell Subsets. Am. J. Pathol..

[139] Menegazzi M., Campagnari R., Bertoldi M., Crupi R., Di Paola R., Cuzzocrea S. (2020). Protective Effect of Epigallocatechin-3-Gallate (EGCG) in Diseases with Uncontrolled Immune Activation: Could Such a Scenario Be Helpful to Counteract COVID-19?. Int. J. Mol. Sci..

[140] Semnani M., Mashayekhi F., Azarnia M., Salehi Z. (2017). Effects of green tea epigallocatechin-3-gallate on the proteolipid protein and oligodendrocyte transcription factor 1 messenger RNA gene expression in a mouse model of multiple sclerosis. Folia Neuropathol..

[141] Herges K., Millward J.M., Hentschel N., Infante-Duarte C., Aktas O., Zipp F. (2011). Neuroprotective Effect of Combination Therapy of Glatiramer Acetate and Epigallocatechin-3-Gallate in Neuroinflammation. PLoS ONE.

[142] Janssen A., Fiebiger S., Bros H., Hertwig L., Romero-Suarez S., Hamann I., Chanvillard C., Bellmann-Strobl J., Paul F., Millward J.M. (2015). Treatment of Chronic Experimental Autoimmune Encephalomyelitis with Epigallocatechin-3-Gallate and Glatiramer Acetate Alters Expression of Heme-Oxygenase-1. PLoS ONE.

[143] Shen P.-X., Li X., Deng S.-Y., Zhao L., Zhang Y.-Y., Deng X., Han B., Yu J., Li Y., Wang Z.-Z. (2021). Urolithin A ameliorates experimental autoimmune encephalomyelitis by targeting aryl hydrocarbon receptor. EBioMedicine.

[144] Han B., Shi L., Bao M.-Y., Yu F.-L., Zhang Y., Lu X.-Y., Wang Y., Li D.-X., Lin J.-C., Jia W. (2024). Dietary ellagic acid therapy for CNS autoimmunity: Targeting on Alloprevotella rava and propionate metabolism. Microbiome.

[145] El-Gohary R.M., Shoeib H.M., Barhoma R.A.E., Badr S.M., Zaher S.M., El Gheit R.E.A., Elshora O.A., Elamly M.H., Magar M.R., Abdelkader G.E. (2026). Modulating miRNA-367-3p Expression by Kaempferol Alleviates Experimental Autoimmune Encephalomyelitis: Targeting Fpn1-Dependent Ferroptosis and cAMP/CREB/CNTF Signaling. Neurochem Res..

[146] Lovera J., Ramos A., Devier D., Garrison V., Kovner B., Reza T., Koop D., Rooney W., Foundas A., Bourdette D. (2015). Polyphenon E, non-futile at neuroprotection in multiple sclerosis but unpredictably hepatotoxic: Phase I single group and phase II randomized placebo-controlled studies. J. Neurol. Sci..

[147] Mähler A., Steiniger J., Bock M., Klug L., Parreidt N., Lorenz M., Zimmermann B.F., Krannich A., Paul F., Boschmann M. (2015). Metabolic response to epigallocatechin-3-gallate in relapsing-remitting multiple sclerosis: A randomized clinical trial. Am. J. Clin. Nutr..

[148] Bellmann-Strobl J., Paul F., Wuerfel J., Dörr J., Infante-Duarte C., Heidrich E., Körtgen B., Brandt A., Pfüller C., Radbruch H. (2021). Epigallocatechin Gallate in Relapsing-Remitting Multiple Sclerosis. Neurol. Neuroimmunol. Neuroinflamm..

[149] Rust R., Chien C., Scheel M., Brandt A.U., Dörr J., Wuerfel J., Klumbies K., Zimmermann H., Lorenz M., Wernecke K.-D. (2021). Epigallocatechin Gallate in Progressive MS. Neurol. Neuroimmunol. Neuroinflamm..

[150] Benlloch M., Ballester M.C., Drehmer E., Platero J.L., Carrera-Juliá S., López-Rodríguez M.M., Ceron J.J., Tvarijonaviciute A., Navarro M.Á., Moreno M.L. (2020). Possible Reduction of Cardiac Risk after Supplementation with Epigallocatechin Gallate and Increase of Ketone Bodies in the Blood in Patients with Multiple Sclerosis. A Pilot Study. Nutrients.

[151] Platero J.L., Cuerda-Ballester M., Ibáñez V., Sancho D., Lopez-Rodríguez M.M., Drehmer E., Ortí J.E.d.l.R. (2020). The Impact of Coconut Oil and Epigallocatechin Gallate on the Levels of IL-6, Anxiety and Disability in Multiple Sclerosis Patients. Nutrients.

[152] Karegar S.J., Aryaeian N., Hajiluian G., Suzuki K., Shidfar F., Salehi M., Ashtiani B.H., Farhangnia P., Delbandi A.-A. (2023). Ellagic acid effects on disease severity, levels of cytokines and T-bet, RORγt, and GATA3 genes expression in multiple sclerosis patients: A multicentral-triple blind randomized clinical trial. Front. Nutr..

[153] Karegar S.J., Mirzaei A., Hajiluian G., Aryaeian N., Shidfar F., Arabgol S., Khosravifar S., Moradiani M., Aghcheli M., Kayyal M. (2025). Ellagic acid favorable effects on fatigue, depression and anxiety in patients with multiple sclerosis and moderate disability: A randomized clinical trial. Mult. Scler. J. Exp. Transl. Clin..

[154] Massa J., O’Reilly E., Munger K., Ascherio A. (2013). Caffeine and alcohol intakes have no association with risk of multiple sclerosis. Mult. Scler. J..

[155] Lu H., Wu P.-F., Zhang W., Xia K. (2020). Coffee consumption is not associated with risk of multiple sclerosis: A Mendelian randomization study. Mult. Scler. Relat. Disord..

[156] Hedström A.K., Mowry E.M., A Gianfrancesco M., Shao X., A Schaefer C., Shen L., Olsson T., Barcellos L.F., Alfredsson L. (2016). High consumption of coffee is associated with decreased multiple sclerosis risk; results from two independent studies. J. Neurol. Neurosurg. Psychiatry.

[157] Ponsonby A.-L., Lucas R.M., Dear K., van der Mei I., Taylor B., Chapman C., Coulthard A., Dwyer T., Kilpatrick T.J., McMichael A.J. (2013). The physical anthropometry, lifestyle habits and blood pressure of people presenting with a first clinical demyelinating event compared to controls: The Ausimmune study. Mult. Scler. J..

[158] Dastoorpoor M., Nabavi S.M., Majdinasab N., Zare Javid A., Ahmadi Angali K., Seyedtabib M. (2023). A case–control study of drinking beverages and the risk of multiple sclerosis in Iran. J. Health Popul. Nutr..

[159] Ivashynka A., Leone M.A., Barizzone N., Cucovici A., Cantello R., Vecchio D., Zuccalà M., Pizzino A., Copetti M., D’ALfonso S. (2022). The impact of lifetime coffee and tea loads on Multiple Sclerosis severity. Clin. Nutr. ESPEN.

[160] Go A.S., Mozaffarian D., Roger V.L., Benjamin E.J., Berry J.D., Blaha M.J., Dai S., Ford E.S., Fox C.S., Franco S. (2014). Heart Disease and Stroke Statistics—2014 Update. Circulation.

[161] Voskoboinik A., Koh Y., Kistler P.M. (2019). Cardiovascular effects of caffeinated beverages. Trends Cardiovasc. Med..

[162] Chieng D., Kistler P.M. (2022). Coffee and tea on cardiovascular disease (CVD) prevention. Trends Cardiovasc. Med..

[163] Yarmolinsky J., Gon G., Edwards P. (2015). Effect of tea on blood pressure for secondary prevention of cardiovascular disease: A systematic review and meta-analysis of randomized controlled trials. Nutr. Rev..

[164] Onakpoya I., Spencer E., Heneghan C., Thompson M. (2014). The effect of green tea on blood pressure and lipid profile: A systematic review and meta-analysis of randomized clinical trials. Nutr. Metab. Cardiovasc. Dis..

[165] Khalesi S., Sun J., Buys N., Jamshidi A., Nikbakht-Nasrabadi E., Khosravi-Boroujeni H. (2014). Green tea catechins and blood pressure: A systematic review and meta-analysis of randomised controlled trials. Eur. J. Nutr..

[166] Duffy S.J., Keaney J.F., Holbrook M., Gokce N., Swerdloff P.L., Frei B., Vita J.A. (2001). Short- and Long-Term Black Tea Consumption Reverses Endothelial Dysfunction in Patients with Coronary Artery Disease. Circulation.

[167] Sano J., Inami S., Seimiya K., Ohba T., Sakai S., Takano T., Mizuno K. (2004). Effects of Green Tea Intake on the Development of Coronary Artery Disease. Circ. J..

[168] Hirano R., Momiyama Y., Takahashi R., Taniguchi H., Kondo K., Nakamura H., Ohsuzu F. (2002). Comparison of green tea intake in Japanese patients with and without angiographic coronary artery disease. Am. J. Cardiol..

[169] Konstantinidi M., Koutelidakis A.E. (2019). Functional Foods and Bioactive Compounds: A Review of Its Possible Role on Weight Management and Obesity’s Metabolic Consequences. Medicines.

[170] Chen I.-J., Liu C.-Y., Chiu J.-P., Hsu C.-H. (2016). Therapeutic effect of high-dose green tea extract on weight reduction: A randomized, double-blind, placebo-controlled clinical trial. Clin. Nutr..

[171] Momose Y., Maeda-Yamamoto M., Nabetani H. (2016). Systematic review of green tea epigallocatechin gallate in reducing low-density lipoprotein cholesterol levels of humans. Int. J. Food Sci. Nutr..

[172] Huang S., Li J., Wu Y., Ranjbar S., Xing A., Zhao H., Wang Y., Shearer G.C., Bao L., Lichtenstein A.H. (2018). Tea Consumption and Longitudinal Change in High-Density Lipoprotein Cholesterol Concentration in Chinese Adults. J. Am. Heart Assoc..

[173] Dludla P.V., Nkambule B.B., Mazibuko-Mbeje S.E., Nyambuya T.M., Orlando P., Silvestri S., Marcheggiani F., Cirilli I., Ziqubu K., Ndevahoma F. (2021). Tea consumption and its effects on primary and secondary prevention of coronary artery disease: Qualitative synthesis of evidence from randomized controlled trials. Clin. Nutr. ESPEN.

[174] Dower J.I., Geleijnse J.M., Gijsbers L., Zock P.L., Kromhout D., Hollman P.C. (2015). Effects of the pure flavonoids epicatechin and quercetin on vascular function and cardiometabolic health: A randomized, double-blind, placebo-controlled, crossover trial. Am. J. Clin. Nutr..

[175] Saito E., Inoue M., Sawada N., Shimazu T., Yamaji T., Iwasaki M., Sasazuki S., Noda M., Iso H., Tsugane S. (2015). Association of green tea consumption with mortality due to all causes and major causes of death in a Japanese population: The Japan Public Health Center-based Prospective Study (JPHC Study). Ann. Epidemiol..

[176] Kuriyama S., Shimazu T., Ohmori K., Kikuchi N., Nakaya N., Nishino Y., Tsubono Y., Tsuji I. (2006). Green Tea Consumption and Mortality Due to Cardiovascular Disease, Cancer, and All Causes in Japan. JAMA.

[177] Arab L., Liu W., Elashoff D. (2009). Green and Black Tea Consumption and Risk of Stroke. Stroke.

[178] Shen L., Song L., Ma H., Jin C., Wang J., Xiang M. (2012). Tea consumption and risk of stroke: A dose-response meta-analysis of prospective studies. J. Zhejiang Univ. Sci. B.

[179] Azami S., Forouzanfar F. (2023). Therapeutic potentialities of green tea (*Camellia sinensis*) in ischemic stroke: Biochemical and molecular evidence. Metab. Brain Dis..

[180] Gundimeda U., McNeill T.H., Elhiani A.A., Schiffman J.E., Hinton D.R., Gopalakrishna R. (2012). Green Tea Polyphenols Precondition against Cell Death Induced by Oxygen-Glucose Deprivation via Stimulation of Laminin Receptor, Generation of Reactive Oxygen Species, and Activation of Protein Kinase Cϵ. J. Biol. Chem..

[181] Kim S.-R., Seong K.-J., Kim W.-J., Jung J.-Y. (2022). Epigallocatechin Gallate Protects against Hypoxia-Induced Inflammation in Microglia via NF-κB Suppression and Nrf-2/HO-1 Activation. Int. J. Mol. Sci..

[182] Zhang J.-C., Xu H., Yuan Y., Chen J.-Y., Zhang Y.-J., Lin Y., Yuan S.-Y. (2017). Delayed Treatment with Green Tea Polyphenol EGCG Promotes Neurogenesis After Ischemic Stroke in Adult Mice. Mol. Neurobiol..

[183] Park D.-J., Kang J.-B., Shah M.-A., Koh P.-O. (2021). Epigallocatechin Gallate Alleviates Down-Regulation of Thioredoxin in Ischemic Brain Damage and Glutamate-Exposed Neuron. Neurochem. Res..

[184] Lin M.-C., Liu C.-C., Lin Y.-C., Hsu C.-W. (2022). Epigallocatechin Gallate Modulates Essential Elements, Zn/Cu Ratio, Hazardous Metal, Lipid Peroxidation, and Antioxidant Activity in the Brain Cortex during Cerebral Ischemia. Antioxidants.

[185] You Y.-P. (2016). Epigallocatechin Gallate Extends the Therapeutic Window of Recombinant Tissue Plasminogen Activator Treatment in Ischemic Rats. J. Stroke Cerebrovasc. Dis..

[186] Yao R.-Q., Qi D.-S., Yu H.-L., Liu J., Yang L.-H., Wu X.-X. (2012). Quercetin Attenuates Cell Apoptosis in Focal Cerebral Ischemia Rat Brain Via Activation of BDNF–TrkB–PI3K/Akt Signaling Pathway. Neurochem. Res..

[187] Lee J.-K., Kwak H.-J., Piao M.-S., Jang J.-W., Kim S.-H., Kim H.-S. (2011). Quercetin reduces the elevated matrix metalloproteinases-9 level and improves functional outcome after cerebral focal ischemia in rats. Acta Neurochir..

[188] Kakuda T., Yanase H., Utsunomiya K., Nozawa A., Unno T., Kataoka K. (2000). Protective effect of γ-glutamylethylamide (theanine) on ischemic delayed neuronal death in gerbils. Neurosci. Lett..

[189] Egashira N., Hayakawa K., Osajima M., Mishima K., Iwasaki K., Oishi R., Fujiwara M. (2007). Involvement of GABAA Receptors in the Neuroprotective Effect of Theanine on Focal Cerebral Ischemia in Mice. J. Pharmacol. Sci..

[190] Wang X.-H., You Y.-P. (2017). Epigallocatechin Gallate Extends Therapeutic Window of Recombinant Tissue Plasminogen Activator Treatment for Brain Ischemic Stroke: A Randomized Double-Blind and Placebo-Controlled Trial. Clin. Neuropharmacol..

[191] ByByron S.A., Tran N.L., Halperin R.F., Phillips J.J., Kuhn J.G., de Groot J.F., Colman H., Ligon K.L., Wen P.Y., Cloughesy T.F. (2018). Prospective Feasibility Trial for Genomics-Informed Treatment in Recurrent and Progressive Glioblastoma. Clin. Cancer Res..

[192] Bleeker F.E., Molenaar R.J., Leenstra S. (2012). Recent advances in the molecular understanding of glioblastoma. J. Neurooncol..

[193] Grube S., Ewald C., Kögler C., Lawson McLean A., Kalff R., Walter J. (2018). Achievable Central Nervous System Concentrations of the Green Tea Catechin EGCG Induce Stress in Glioblastoma Cells in Vitro. Nutr. Cancer.

[194] Mukherjee S., Baidoo J.N.E., Sampat S., Mancuso A., David L., Cohen L.S., Zhou S., Banerjee P. (2018). Liposomal TriCurin, A Synergistic Combination of Curcumin, Epicatechin Gallate and Resveratrol, Repolarizes Tumor-Associated Microglia/Macrophages, and Eliminates Glioblastoma (GBM) and GBM Stem Cells. Molecules.

[195] Santos B.L., Oliveira M.N., Coelho P.L., Pitanga B.P., da Silva A.B., Adelita T., Silva V.D.A., Costa M.d.F., El-Bachá R.S., Tardy M. (2015). Flavonoids suppress human glioblastoma cell growth by inhibiting cell metabolism, migration, and by regulating extracellular matrix proteins and metalloproteinases expression. Chem. Biol. Interact..

[196] Lin C., Shen S., Chien C., Yang L., Shia L., Chen Y. (2010). 12- *O* -tetradecanoylphorbol-13-acetate-induced invasion/migration of glioblastoma cells through activating PKCα/ERK/NF-κB-dependent MMP-9 expression. J. Cell. Physiol..

[197] Pedra N.S., Bona N.P., de Aguiar M.S.S., Spohr L., Alves F.L., Santos F.d.S.d., Saraiva J.T., Stefanello F.M., Braganhol E., Spanevello R.M. (2022). Impact of gallic acid on tumor suppression: Modulation of redox homeostasis and purinergic response in in vitro and a preclinical glioblastoma model. J. Nutr. Biochem..

[198] Wang D., Chen Q., Tan Y., Liu B., Liu C. (2017). Ellagic acid inhibits human glioblastoma growth in vitro and in vivo. Oncol. Rep..

[199] Cetin A., Biltekin B., Ozevren H. (2022). Antitumor activity of irinotecan with ellagic acid in C6 glioma cells. Rev. Assoc. Med. Bras..

[200] Cetin A., Biltekin B. (2019). Ellagic acid enhances antitumor efficacy of temozolomide in an in vitro glioblastoma model. Turk. Neurosurg..

[201] Çetin A., Biltekin B., Degirmencioglu S. (2019). Ellagic Acid Enhances the Antitumor Efficacy of Bevacizumab in an In Vitro Glioblastoma Model. World Neurosurg..

[202] Chang Y., Kim J., Park H., Choi H., Kim J. (2020). Modelling neurodegenerative diseases with 3D brain organoids. Biol. Rev..

[203] Ineichen B.V., Furrer E., Grüninger S.L., Zürrer W.E., Macleod M.R. (2024). Analysis of animal-to-human translation shows that only 5% of animal-tested therapeutic interventions obtain regulatory approval for human applications. PLoS Biol..

[204] Larriba-González T., García-Martín M., Ojeda-Hernández D.D., Rincón-Cerrada P., Martín-Blanco L., Benito-Martín M.S., Selma-Calvo B., de la Fuente-Martín S., A Matias-Guiu J., Matias-Guiu J. (2025). Modeling neurodegenerative diseases with brain organoids: From development to disease applications. Front. Cell Dev. Biol..

[205] Zhang J. (2012). Encapsulated nanoepigallocatechin-3-gallate and elemental selenium nanoparticles as paradigms for nanochemoprevention. Int. J. Nanomed..

[206] Tang Q., Liu K., Yue C., Luo L., Zeng L., Wu Z. (2023). *CsXDH1* gene promotes caffeine catabolism induced by continuous strong light in tea plant. Hortic. Res..

[207] Li J., Xiao Y., Zhou X., Liao Y., Wu S., Chen J., Qian J., Yan Y., Tang J., Zeng L. (2022). Characterizing the cultivar-specific mechanisms underlying the accumulation of quality-related metabolites in specific Chinese tea (*Camellia sinensis*) germplasms to diversify tea products. Food Res. Int..

[208] Peng P., Wang L., Shu G., Li J., Chen L. (2020). Nutrition and aroma challenges of green tea product as affected by emerging superfine grinding and traditional extraction. Food Sci. Nutr..

[209] Li K., Teng C., Min Q. (2020). Advanced Nanovehicles-Enabled Delivery Systems of Epigallocatechin Gallate for Cancer Therapy. Front Chem..

[210] Tang D.-W., Yu S.-H., Ho Y.-C., Huang B.-Q., Tsai G.-J., Hsieh H.-Y., Sung H.-W., Mi F.-L. (2013). Characterization of tea catechins-loaded nanoparticles prepared from chitosan and an edible polypeptide. Food Hydrocoll..

[211] Daraee H., Etemadi A., Kouhi M., Alimirzalu S., Akbarzadeh A. (2016). Application of liposomes in medicine and drug delivery. Artif. Cells Nanomed. Biotechnol..

[212] Mehmood S., Maqsood M., Mahtab N., Khan M.I., Sahar A., Zaib S., Gul S. (2022). Epigallocatechin gallate: Phytochemistry, bioavailability, utilization challenges, and strategies. J. Food Biochem..

[213] Goktas Z., Zu Y., Abbasi M., Galyean S., Wu D., Fan Z., Wang S. (2020). Recent Advances in Nanoencapsulation of Phytochemicals to Combat Obesity and Its Comorbidities. J. Agric. Food Chem..

[214] Pattni B.S., Chupin V.V., Torchilin V.P. (2015). New Developments in Liposomal Drug Delivery. Chem. Rev..

[215] Beloqui A., Solinís M.Á., Rodríguez-Gascón A., Almeida A.J., Préat V. (2016). Nanostructured lipid carriers: Promising drug delivery systems for future clinics. Nanomedicine.

[216] Clevers H. (2016). Modeling Development and Disease with Organoids. Cell.

[217] Quadrato G., Nguyen T., Macosko E.Z., Sherwood J.L., Min Yang S., Berger D.R., Maria N., Scholvin J., Goldman M., Kinney J.P. (2017). Cell diversity and network dynamics in photosensitive human brain organoids. Nature.

[218] Trujillo C.A., Gao R., Negraes P.D., Gu J., Buchanan J., Preissl S., Wang A., Wu W., Haddad G.G., Chaim I.A. (2019). Complex Oscillatory Waves Emerging from Cortical Organoids Model Early Human Brain Network Development. Cell Stem Cell.

[219] Fontanella F., D’Alessandro T., Nardone E., De Stefano C., Vicidomini C., Roviello G.N. (2026). Artificial Intelligence for Natural Products Drug Discovery in Neurodegenerative Therapies: A Review. Biomolecules.

[220] Mullowney M.W., Duncan K.R., Elsayed S.S., Garg N., van der Hooft J.J.J., Martin N.I., Meijer D., Terlouw B.R., Biermann F., Blin K. (2023). Artificial intelligence for natural product drug discovery. Nat. Rev. Drug Discov..

[221] Yu Y., Zhou M., Long X., Yin S., Hu G., Yang X., Jian W., Yu R. (2023). Study on the mechanism of action of colchicine in the treatment of coronary artery disease based on network pharmacology and molecular docking technology. Front. Pharmacol..

[222] Bai G., Pan Y., Zhang Y., Li Y., Wang J., Wang Y., Teng W., Jin G., Geng F., Cao J. (2023). Research advances of molecular docking and molecular dynamic simulation in recognizing interaction between muscle proteins and exogenous additives. Food Chem..

[223] Lemkul J.A., Bevan D.R. (2012). The Role of Molecular Simulations in the Development of Inhibitors of Amyloid β-Peptide Aggregation for the Treatment of Alzheimer’s Disease. ACS Chem. Neurosci..

